# Ancient genomes reveal complex patterns of population movement, interaction, and replacement in sub-Saharan Africa

**DOI:** 10.1126/sciadv.aaz0183

**Published:** 2020-06-12

**Authors:** Ke Wang, Steven Goldstein, Madeleine Bleasdale, Bernard Clist, Koen Bostoen, Paul Bakwa-Lufu, Laura T. Buck, Alison Crowther, Alioune Dème, Roderick J. McIntosh, Julio Mercader, Christine Ogola, Robert C. Power, Elizabeth Sawchuk, Peter Robertshaw, Edwin N. Wilmsen, Michael Petraglia, Emmanuel Ndiema, Fredrick K. Manthi, Johannes Krause, Patrick Roberts, Nicole Boivin, Stephan Schiffels

**Affiliations:** 1 Department of Archaeogenetics, Max Planck Institute for the Science of Human History, Jena, Germany.; 2 Department of Archaeology, Max Planck Institute for the Science of Human History, Jena, Germany.; 3 UGent Centre for Bantu Studies, Department of Languages and Cultures, Ghent University, Ghent, Belgium.; 4 Institut des Mondes Africains, Paris, France.; 5 Institut des Musées Nationaux du Congo, Kinshasa, Democratic Republic of Congo.; 6 Department of Archaeology, University of Cambridge, Cambridge, UK.; 7 Department of Anthropology, University of California, Davis, Davis, CA, USA.; 8 School of Social Science, University of Queensland, St Lucia, Brisbane, QLD 4072, Australia.; 9 Department of History, Cheikh Anta Diop University, Dakar, Senegal.; 10 Department of Anthropology, Yale University, New Haven, CT, USA.; 11 Department of Archaeology and Anthropology, University of Calgary, Calgary, Alberta, Canada.; 12 Department of Earth Sciences, National Museums of Kenya, Nairobi, Kenya.; 13 Institute for Pre- and Protohistoric Archaeology and Archaeology of the Roman Provinces, Ludwig-Maximilians-University Munich, Munich, Germany.; 14 Department of Anthropology, Stony Brook University, Stony Brook, NY, USA.; 15 Department of Anthropology, California State University, San Bernardino, San Bernardino, CA, USA.; 16 University of Texas-Austin, Austin, TX, USA.; 17 Witwatersrand University, Johannesburg, Republic of South Africa.; 18 Department of Anthropology, Smithsonian Institution, Washington, DC, USA.

## Abstract

Africa hosts the greatest human genetic diversity globally, but legacies of ancient population interactions and dispersals across the continent remain understudied. Here, we report genome-wide data from 20 ancient sub-Saharan African individuals, including the first reported ancient DNA from the DRC, Uganda, and Botswana. These data demonstrate the contraction of diverse, once contiguous hunter-gatherer populations, and suggest the resistance to interaction with incoming pastoralists of delayed-return foragers in aquatic environments. We refine models for the spread of food producers into eastern and southern Africa, demonstrating more complex trajectories of admixture than previously suggested. In Botswana, we show that Bantu ancestry post-dates admixture between pastoralists and foragers, suggesting an earlier spread of pastoralism than farming to southern Africa. Our findings demonstrate how processes of migration and admixture have markedly reshaped the genetic map of sub-Saharan Africa in the past few millennia and highlight the utility of combined archaeological and archaeogenetic approaches.

## Teaser

New ancient genomes from Africa provide details of the spread of food production across sub-Saharan Africa.

## INTRODUCTION

Africa today hosts enormous linguistic, cultural, and economic diversity. Reconstructing the patterns of population interaction, migration, admixture, and replacement that contributed to this diversity has been a core aim of genetic, archaeological, and linguistic studies for decades (*1*–*4*). As a relatively young field of research, ancient DNA (aDNA) has contributed less to these multidisciplinary efforts than other disciplines, and as a result of the limitations of skeletal and DNA preservation in Africa, aDNA has contributed less to African prehistory than elsewhere. While technical advances, such as the recognition of the petrous part of the temporal bone as a region that preserves high endogenous aDNA (*5*), have begun to change this situation, Africa remains understudied with only 85 ancient genomes published from the continent to date, relative to 3500 from Eurasia.

Previous aDNA studies from Africa have provided insights into population structure before the spread of food production in eastern and southern Africa (*2*, *3*, *6*) and revealed evidence for population turnovers in relation to changes in subsistence strategies in eastern Africa (*4*). Broadly, forager populations sampled between eastern and southern Africa were shown to have formed a continuous genetic cline roughly following geography (*3*). During the Pastoral Neolithic (PN), people related to Chalcolithic and Bronze Age Levantine groups entered eastern Africa and mixed there with individuals related to Later Stone Age foragers and with individuals related to present-day Dinka in what was proposed to have been at least a two-step process (*4*). Ancestry related to present-day Bantu speakers, which is, today, prevalent across sub-Saharan Africa, is absent from most ancient sub-Saharan African genomes analyzed to date.

Here, we report new insights into early population movements and admixture in Africa based on analysis of 20 newly generated ancient sub-Saharan African genomes (Table 1). Our sampling strategy follows a transregional approach to investigating population-level interactions between key groups that were identified previously as being involved in changes of food production strategies: eastern and southern forager groups, eastern African Pastoral Neolithic and Iron Age groups, and Iron Age groups related to present-day Bantu speakers. We sampled individuals from key regions where current models not only predict substantial interaction between foragers, herders, and farmers, particularly in eastern Africa, but also include the first individuals sampled from the Democratic Republic of the Congo (DRC), Botswana, and Uganda. By adding these new ancient genomes derived from archaeological forager and food-producing populations to published ancient and present-day sub-Saharan African genomes, we detect (i) evidence for the contraction of previously widespread and overlapping, deeply diverged forager populations; (ii) indications that the arrival of pastoral populations in eastern Africa resulted from the movement of several discrete groups of herders from northern to eastern Africa; and (iii) evidence for notable geographic diversity in patterns of herder-farmer-forager admixture during the spread of food production. These models are strengthened by integrating the first ancient genomes from the DRC, Botswana, and Uganda, allowing us to extend these multibranch models for the spread of food production across the continent. Data from Botswana also allow us to suggest a dispersal of eastern African pastoralists into southern Africa before the arrival of Bantu-speaking populations as has been previously suggested on the basis of linguistic and modern genetic data (*7*, *8*). Together, the ancient genomic and archaeological data examined here indicate that the economic heterogeneity that is the hallmark of modern Africa resulted from diverse local histories of population admixture, interaction, and avoidance.

**Table 1:** Summary of individuals with successful aDNA from Africa reported in this study. Note 1: Two samples from Lukenya Hill (LUK001 and LUK002) tend out to be genetically the same individual. We merged the genomic data for genetic analyses but report radiocarbon dates for both here. Note 2: The age of samples marked with asterisk is based on the archeological context instead of calibrated radiocarbon date. Aut. Cov., autosomal coverage; MT Cov., mitochondrial coverage.

**ID**	**Population label**	**Archeological** **site**	**Country**	**Archeological** **affiliation**	**Aut.** **Cov.**	**MT** **Cov.**	**Sex**	**Y haplogroup**	**MT** **haplogroup**	**Aut.** **SNPs**	**SNPs** **hit on** **Human** **Origin** **dataset**	**Date** **(calendar** **BP)**	**Uncalibrated** **C14** **dates ± error** **(lab number)**
NYA002	Kenya_ Nyarindi_ 3500BP	Nyarindi Rockshelter	Kenya	Later Stone Age (Kansyore)	0.14	0.23	F	–	L4b2a	124,064	64,785	3555–3375	3253 ± 23 (OxA-37364)
NYA003	Kenya_ Nyarindi_ 3500BP	Nyarindi Rockshelter	Kenya	Later Stone Age (Kansyore)	0.02	0.02	M	E(E-M96,E-P162)	–	18,586	9736	–	–
LUK001	Kenya_ LukenyaHill_ 3500BP	Lukenya Hill, GvJm 202	Kenya	Pastoral Neolithic	0.59	1.41	M	E1b1b1b2b(E- M293,E- CTS10880)	L4b2a2b	495,472	222,439	3610– 3460	3340 ± 23 (OxA-37356), 3296 ± 25 (OxA-37357)
LUK003	Kenya_ LukenyaHill_ 3500BP	Lukenya Hill, GvJm 202	Kenya	Pastoral Neolithic	0.01	0.30	F	–	L0f1	6830	3586	3635– 3475	3359 ± 23 (OxA-37358)
HYR002	Kenya_ HyraxHill_ 2300BP	Hyrax Hill, GrJj25	Kenya	Pastoral Neolithic	0.77	0.52	M	E1b1b1b2b(E- M293,E-M293)	L5a1b	505,972	260,999	2365– 2305	2354 ± 23 (OxA-37352)
MOL001	Kenya_ MoloCave_ 1500BP	Molo Cave, GoJi3	Kenya	Pastoral Neolithic	2.64	5.40	M	E1b1b1b2b(E- M293,E-M293)	L3h1a2a1	886,222	461,756	1415– 1320	1532 ± 21 (OxA-37360)
MOL003	Kenya_ MoloCave_ 1500BP	Molo Cave, GoJi3	Kenya	Pastoral Neolithic	0.06	0.14	F	–	–	57,426	29,700	2110– 1990	2101 ± 22 (OxA-37361)
KPL001	Kenya_ Kakapel_ 3900BP	Kakapel	Kenya	Later Stone Age (Kansyore)	0.92	3.94	M	CT(CT- M168,CT-M5695)	L3i1	572,074	299,181	3974– 3831	3584 ± 28 [SUERC-86057 (GU51350)]
KPL002	Kenya_ Kakapel_ 300BP	Kakapel	Kenya	Later Iron Age/ protohistoric	1.26	78.35	F	–	L2a1f	684,698	363,447	309–145	222 ± 28 [SUERC-86058 (GU51351)]
KPL003	Kenya_ Kakapel_ 900BP	Kakapel	Kenya	Later Iron Age	0.07	63.21	F	–	L2a5	75,113	39,367	910–736	895 ± 28 [SUERC-86059 (GU51352)]
MUN001*	Uganda_ Munsa_ 500BP	Munsa	Uganda	Later Iron Age	0.46	1.57	F	–	L3b1a1	377,332	–	1400– 1600 CE	–
KIN002	Congo_ Kindoki_ 230BP	Kindoki	DR Congo	Protohistoric	0.62	1.46	M	E1b1a1a1d1a2(E- CTS99,E-CTS99)	L1c3a1b	438,125	229,240	295–145	217 ± 20 (OxA-37353)
KIN003	Congo_ Kindoki_ 150BP	Kindoki	DR Congo	Protohistoric	0.02	0.09	M	E(E-M96,E- PF1620)	–	19,691	10,329	285– modern	172 ± 20 (OxA-37354)
KIN004	Congo_ Kindoki_230BP	Kindoki	DR Congo	Protohistoric	0.96	2.01	M	R1b1(R- P25_1,R-M415)	L0a1b1a1	560,376	291,465	305–150	241 ± 20 (OxA-37355)
NGO001	Congo_ NgongoMbata_ 220BP	Ngongo Mbata	DR Congo	Protohistoric	0.42	0.78	M	–	L1c3a	328,389	170,742	295–145	211 ± 21 (OxA-37363)
MTN001	Congo_ MatangaiTuru_ 750BP	Matangai Turu Northwest	DR Congo	Iron Age forager	0.06	0.33	F	–	–	52,012	28,452	795–690	871 ± 21 (OxA-37362)
NQO002*	Botswana_ Nqoma_ 900BP	Nqoma	Botswana	Early Iron Age	0.02	0.60	F	–	L2a1f	14,189	7,587	700- 1090 CE	–
TAU001*	Botswana_ Taukome_ 1100BP	Taukome	Botswana	Early Iron Age	0.09	5.82	M	E1b1a1(E- M2,E-Z1123)	L0d3b1	79,261	42,998	900- 1000 CE	–
XAR001*	Botswana_ Xaro_ 1400BP	Xaro	Botswana	Early Iron Age	3.64	37.94	M	E1b1a1a1c1a	L3e1a2	939,378	494,074	700- 1000 CE	–
XAR002*	Botswana_ Xaro_ 1400BP	Xaro	Botswana	Early Iron Age	1.36	172.94	M	E1b1b1b2b(E- M293,E- CTS10880)	L0k1a2	703,295	375,283	700- 1000 CE	–

## RESULTS

### New aDNA from Africa

**Fig. 1:**
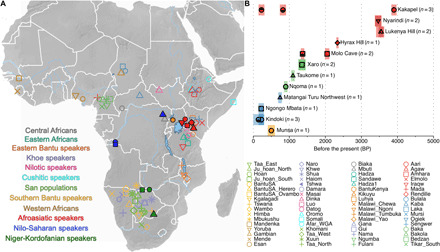
Basic information of newly reported ancient genomes. (**A**) Approximate locations of new samples and published present-day modern African populations. Same legend scheme applies to the principal components analysis (PCA) plot in Fig. 2. (**B**) C14 dates after calibration. Samples from Botswana (green) and Uganda (orange) are based on archaeological context dates rather than accelerator mass spectrometry (AMS) measurements.

We generated new genome-wide data from 20 ancient sub-Saharan African individuals (Table 1 and table S1), after screening skeletal material from 57 individuals (table S2). We evaluated the authenticity of aDNA for all screened samples based on characteristic cytosine-to-thymine deamination at the end of aDNA fragments and performed in-solution enrichment on mitochondria and 1.2 million autosomal single-nucleotide polymorphisms (SNPs) for 23 samples (two did not yield enough data after capture, and two samples were from the same individual) with endogenous DNA content above 0.1%. The successful samples include 5 individuals from the DRC [~795–200 before the present (BP)], 4 from Botswana (~1300–1000 BP), 1 from Uganda (~400–600 BP), and 10 from southern Kenya (~3900–300 BP), of which 3 are associated with eastern African foraging traditions, 5 with Pastoral Neolithic contexts, and 2 from the Iron Age. We combined these newly reported ancient genomes with previously published ancient African genomes (*2*–*4*, *9*–*11*), together with genomes from 584 individuals from 59 contemporary African populations (*1*, *12*), 44 high-coverage genomes from 22 African Indigenous populations (*13*), and 300 high-coverage genomes from 142 worldwide populations (*14*). The ages of the newly reported ancient individuals and their approximate sample locations are shown in Fig. 1. We examined the contamination level for all samples according to mitochondrial contamination estimates (*15*, *16*) and X chromosome contamination in males (table S1) (*17*). We also report mitochondrial haplogroups of each sample and Y chromosome haplogroups for most male samples (Table 1). We analyzed pairwise genetic similarities between all individuals and found that while NYA002 and NYA003 are consistent with being second-degree relatives, all other pairs are unrelated (see Materials and Methods).

### Contraction of previously overlapping hunter-gatherer ancestries

**Fig. 2:**
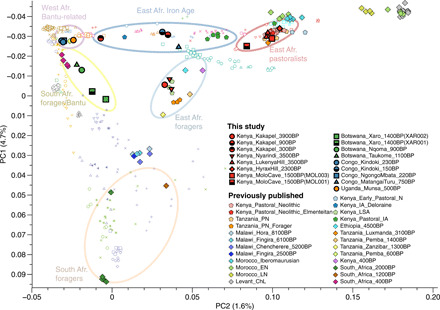
PCA of ancient genomic data analyzed in this and previous studies, together with published modern genetic data. Modern populations shown are detailed in the legend of Fig. 1 and fig. S1. A separate PCA with only present-day populations is shown in fig. S1A. Color circles highlight the key groups discussed in this paper and summarized in Fig. 3A.

**Fig. 3:**
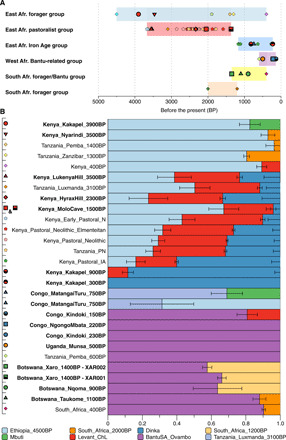
Admixture history of ancient African populations. (**A**) Overview of coexistence of distinct African ancestries through time drawing on currently available ancient genomes. (**B**) Ancestral components of ancient African groups/individuals according to qpAdm. We order ancient groups in the same order shown in (A) and highlight newly reported genetic groups/individuals in bold. *P* values and estimated ancestral proportions can be found in table S3.

We used principal components analysis (PCA) and model-based clustering to characterize the genetic relationship between our ancient individuals and published ancient and present-day African individuals (*1*–*4*, *9*–*14*). We find that our eight Kenyan samples, spanning 3900 to 1500 BP, form two clusters in PCA (Fig. 2), confirmed using ADMIXTURE (fig. S1) (*18*). Cluster 1 (named “east African foragers” in Fig. 2) consists of the new group/individual Kenya_Nyarindi_3500BP and Kenya_Kakapel_3900BP, as well as published data from Tanzania_Pemba_1400BP, Tanzania_Zanzibar_1400BP, and Kenya_400BP (Fig. 2). Cluster 2 (named “east African pastoralists”) includes the new Kenyan samples with eastern African pastoralist-related ancestry. Individuals from cluster 1 show high genetic similarity to the 4500-BP hunter-gatherer from the Mota site in Ethiopia (*9*), as well as previously described ancient foragers from eastern Africa (*3*, *4*). We tested which ancestries other than Ethiopia_4500BP are present in these individuals although statistics of the form *f4* (ancient group, Ethiopia_4500BP; X, chimpanzee), which tests whether any other group X is more closely related to either our ancient individuals or Ethiopia_4500BP (the chimpanzee genome is required for technical reasons as an outgroup to all humans). Among the groups/individuals in this cluster (fig. S2), Kenya_Nyarindi_3500BP and Tanzania_Pemba_1400BP do not demonstrate significant genetic affinity to any other group that we tested here, while Kenya_Kakapel_3900BP shows significant genetic affinity with the Mbuti, a present-day group of Central African hunter-gatherers. In the same test, Tanzania_Zanzibar_1300BP has excess affinity with South_Africa_2000BP, as reported previously (*3*), and Kenya_400BP presents extra affinity with present-day west Eurasian people (*3*). We further characterized genetic ancestry components of these ancient African individuals through qpAdm (*19*), a method to estimate ancestry proportions related to specified source populations. We found Kenya_Kakapel_3900BP has 18 ± 6% Mbuti-related ancestry, and the published Kenya_400BP has 11 ± 3% ancestry related to ancient Levantine individuals (Fig. 3 and table S3), which likely reflects a gene pool present more broadly in ancient northeastern Africa and the Levant, as identified in ancient (*11*, *20*) and present-day northeastern African populations. These additional ancestral contributions are also seen on the PCA (Fig. 2) by their positioning relative to Ethiopia_4500BP. Modeling with qpAdm also suggests a small ancestry component related to southern African San in Kenya_Nyarindi_3500BP (models including San improve the fit significantly, but the resulting *P* value is still low, at *P* = 0.002).

Overall, these data point to eastern Africa as a nexus of population-level interactions between groups with ancestries associated with western, southern, and eastern African foragers. Deep divergences between these ancestries suggest either that admixture was minimal over a long period or that it occurred relatively recently. This poses interesting possibilities for more dynamic expansion and contraction of ancient African hunter-gatherer populations than have been postulated to date. Kenya_Kakapel_3900BP belongs to an archaeological fisher-forager group extending from Lake Victoria well into Uganda, and so the Mbuti-associated ancestry in this individual could be explained by ephemeral interactions between groups whose ranges overlapped when rainforest systems were more extensive in the early Holocene wet phase (*21*). Additional archaeological data from the region are needed to test this hypothesis.

Persistent detection of low levels of San-affiliated ancestry among ancient eastern African individuals is more difficult to explain. One possibility is ongoing interactions with an as-of-yet undetected hunter-gatherer population whose ancestry is primarily shared with the modern San. Another possibility is that the San-related ancestry reflects an earlier, wider distribution of African foragers stretching from southern to eastern Africa, which existed before Mid to Late Holocene migrations of farmers and herder*s* (*3*). Linguistic and genetic parallels between eastern and southern African forager groups using click consonants make it tempting to hypothesize the presence of an early, widely distributed click language-speaking population (*1*, *22*), but there is no phylolinguistic evidence for a direct connection between these language groups (*23*).

### Complex spread of pastoralism to eastern Africa

Cluster 2 of the Kenyan samples on the PCA (Fig. 2), with east African pastoralist-related ancestry, includes the newly reported groups/individuals from sites of the Savanna Pastoral Neolithic tradition in South Kenya: Kenya_LukenyaHill_3500BP, Kenya_HyraxHill_2300BP, and Kenya_MoloCave_1500BP, which fall into the beginning, middle, and end, respectively, of the Pastoral Neolithic period in Kenya, as well as a published ancient genome from Tanzania, Tanzania_Luxmanda_3100BP (*3*), and other published Pastoral Neolithic genomes from eastern Africa (*4*). These samples show remarkable continuity of ancestry across a time span of 2000 years, presenting similar genetic profiles in PCA and clustering analysis (Fig. 2 and fig. S1).

On the basis of previous models for Tanzania_Luxmanda_3100BP (*3*), we first applied two-way ancestry models in qpAdm using Ethiopia_ 4500BP and a group of ancient Levantine individuals (*24*), which we take as the closest available proxy for ancient northeastern African ancestry (*10*, *11*), as sources. Consistent with the findings of a previous aDNA study (*4*), we found this model to be insufficient (Fig. 3 and table S3) and demonstrate that an additional genetic component related to the present-day Dinka (a Nilotic-speaking group from South Sudan) is necessary to fit the data. In addition to qpAdm, we confirmed this affinity using a customized *f4* test (see Materials and Methods and fig. S3). In our final three-way model, which is qualitatively similar to the model proposed in (*4*), we find 33 ± 11% and 24 ± 10% Dinka-related ancestry in Kenya_HyraxHill_2300BP and Kenya_LukenyaHill_3500BP, respectively, and lower proportions in Kenya_MoloCave_1500BP and Tanzania_Lxumanda_3100BP (Fig. 3 and table S3).

While the estimated proportions of Levantine-related ancestry in all samples are rather constant (around 30 to 40%), we find that both the proportion of east African forager-related ancestry, as well as of Dinka-related ancestry, varies substantially across individuals. An earlier study (*4*) concluded that admixture between pioneering herders with Levantine-related ancestry and eastern African hunter-gatherers primarily occurred before their arrival in southern Kenya. However, our data suggest that periodic admixture between herders and hunter-gatherers, or populations predominantly carrying ancestry derived from them, may have continued into the PN. In particular, the newly reported 1500-BP individuals from Molo Cave carry 50% or more forager-related ancestry, and less Dinka-related ancestry, than observed in all other sequenced Pastoral Neolithic individuals (*4*). A model of repeated interaction between foragers and herders is further supported by admixture date estimates using linkage disequilibrium decay, which suggest that admixture dates between ancestry related to Chalcolithic Levant (*24*) and to Ethiopia_4500BP range from a few hundred to a few thousand years before the time of death of the individuals, with no clear correlation between admixture age and age of sample (fig. S4), inconsistent with a simple model of admixture, but suggesting either multiple events, or strong population structure preventing homogenization of ancestries over a long time period. Despite only minimal archaeological evidence for the persistence of autochthonous hunter-gatherers in the Central Rift Valley this late in the PN (*25*), these genetic results suggest that communities with high or unadmixed hunter-gatherer–related ancestry continued to live alongside communities with high or unadmixed Pastoral-Neolithic related ancestry until nearly the Iron Age, leaving prominent genetic traces at Molo Cave. It is not yet clear from Molo Cave or other sites whether the timing and pace of admixture reflects adoption of herding by foragers, absorption of foragers into herding groups, or more complex intergroup social dynamics.

Combining evidence from both eastern African genetic clusters, we document very different patterns of interaction and admixture from sampled individuals along the eastern African and Lake Victoria shores relative to the patterns in the Central Rift. Near lake and ocean coasts, we see little evidence for pastoralist admixture into forager individuals [e.g., Kenya_Nyarindi_3500BP and two previously sampled individuals from Zanzibar (*3*)]. Our analysis also demonstrates that the recently published individual from the cave site of Panga ya Saidi in coastal Kenya [Kenya_400BP (*3*)] similarly retains a predominantly eastern African forager ancestry, with only a small Levantine-related component. This is the exact opposite of the pattern observed in individuals around the Central Rift, where pastoralist-mediated, Levantine-related ancestry spread rapidly. It may be that delayed-return foragers in stable coastal and lacustrine environments were more demographically numerous and/or resistant to interactions with incoming food producers than other hunter-gatherers.

While our data support the three-component model for the Pastoral Neolithic (*4*), our findings suggest greater complexity than initially proposed for the admixture of existing and incoming populations in this period. The fact that both Dinka-related ancestry and eastern African forager-related ancestry varies substantially in our samples and previously published samples suggests that the spread of herding either involved complex population structure maintained over a long time period or prevented homogenization of these ancestries, or multiple population movements with regionally distinct trajectories of interaction and admixture. This adds increasing resolution to proposed diversity of populations that contributed to the “moving frontier” model for herder dispersals in eastern Africa (*4*, *26*). Individuals from Molo Cave, Luxmanda, and Panga ya Saidi furthermore provide evidence that contact with eastern African foragers, who coexisted with food producing people until at least 400 BP (Fig. 3A), was a continuous process, rather than one that occurred only during initial phases of contact.

The data also reveal that this interaction between herders and foragers was very imbalanced, with hunter-gatherer ancestry entering pastoralist populations, but little flow in the other direction. It is not clear what forms of social systems between herders and foragers may have resulted in this one-way admixture. In the past, it has been assumed that low herder population density and high risk of herd loss from epizootic disease would require herders to form closer relationships with local hunter-gatherers who had greater ecological knowledge of the landscape (*27*, *28*). This has been supported by evidence for herder-forager interactions at sites such as Crescent Island (*29*) and Prolonged Drift (*30*). Genetic evidence indicates that if these interactions occurred, then they were more structured and possibly more consistent with ethnographic client-patron relationships (*31*), wherein individuals from hunter-gatherer communities may be slowly integrated into herder societies. It is possible that sex bias due to different social dynamics played a role in the observed asymmetric gene flow between the two groups. While we could not test this explicitly due to insufficient coverage on the X chromosomes, these dynamics have been previously described between foragers in central and southern Africa and Bantu-speaking farmers (*32*).

### Shifts of ancestry during the Iron Age in central and eastern Africa

Three new samples also allowed us to evaluate changes in ancestry during the Iron Age. The Kakapel site in western Kenya, from which we analyzed the 3900-year-old forager above, also featured two Iron Age individuals (Kenya_Kakapel_300BP and Kenya_Kakapel_900BP), which show close genetic affinity to Dinka and other Nilotic-speaking groups (Luo, Datog, and Maasai) using PCA and ADMIXTURE, and also have closer genetic affinity with present-day Bantu speakers than ancient foragers or Pastoral Neolithic individuals (Fig. 2 and figs. S1 and S5).

On the basis of the affinity seen on the PCA, we tested whether Kenya_Kakapel_300BP and Kenya_Kakapel_900BP are genetically similar to the Nilotic-speaking Dinka and Luo and Bantu-speaking Luhya and Kikuyu (all are ethnic groups in modern Kenya, except the Dinka of South Sudan). Using *f4* statistics and qpAdm, we find that Kenya_Kakapel_300BP is similar in ancestry to Dinka, with Luo and Luhya providing marginally fitting models as well (Fig. 3, fig. S5, and table S4). Kenya_Kakapel_900BP also shares close genetic affinity with Dinka but requires an additional small ancestry component (12 ± 3%) from northeastern African/Levantine groups, similar to the ancestry component in early PN herders (Fig. 3 and table S4). We dated this admixture between Dinka- and Levantine-related ancestries in Kenya_Kakapel_900BP to around 500 ± 200 years before the death of that individual, consistent with the onset of the Iron Age in the region. This suggests that the Iron Age population represented by this single individual resulted from admixture between PN-related herders and incoming Nilotic agropastoralists, rather than resulting from a major migration of people with West African–related ancestries.

The notable shift seen in the two Iron Age individuals from the Kakapel site to almost 90 to 100% Nilotic-related ancestry, compared to about 40% during the Pastoral Neolithic, is substantially larger than the increase in Nilotic ancestry seen in previously analyzed eastern African individuals from the Iron Age (*4*). In addition, the absence of ancestry related to present-day Bantu speakers in Kenya_Kakapel_900BP contrasts with the finding of this ancestry in a contemporaneous individual from the site of Deloraine farm in the Central Rift Valley of Kenya (*4*). This shows that patterns of dispersal and admixture in Iron Age eastern Africa resulted in a complex geography of ancestry, with some regions or locations witnessing almost complete replacement from Nilotic-related migrations (*33*), others seeing mixing of diverse peoples (*4*), and yet others demonstrating no admixture from ancestry related to Nilotic or Bantu speakers into recent centuries (as seen in Kenya_400BP).

Previous research associated the increase in Nilotic ancestry during the Iron Age with a so-called “Pastoral Iron Age” based on samples from the Central Rift Valley (*4*). Our findings for the Iron Age, much like our findings for the PN, are consistent with multiple groups with different subsistence systems entering eastern Africa along different geographical routes. While these can broadly be grouped as a single “stage” of population change (*4*), it is increasingly clear that there is greater heterogeneity in the nature of population change within southern Kenya than previously recognized.

A new Iron Age genome from the eastern border of the DRC (Congo_MatangaiTuru_750BP) highlights additional trajectories of forager–food producer interaction as herding and farming spread into Central Africa. The best-fitting model for this individual is one including Ethiopia_4500BP as one source and Pastoral Neolithic as the other (Fig. 3 and table S5). We tested an alternative model with Mbuti instead of Ethiopia_4500BP, which also provided a working fit and which fits a signal seen on PCA (specifically, PC4; see fig. S8), which shows that this individual is shifted toward Mbuti. While the sparse genetic data available for the Matangai Turu individual did not allow us to select between these two models, we highlight that both models indicate PN-related ancestry in a region hitherto unsampled for aDNA. We argue that this finding may reflect continued expansion of Pastoral Neolithic populations, with or without herding, during the Iron Age, possibly related, or in response to, displacement by incoming groups related to Nilotic- and Bantu-speaking populations. We caution that this argument is based on a single individual and more data from the region are necessary to make stronger statements. Our successful aDNA extraction from a rainforest location shows that this is possible.

A single sample from Munsa, Uganda, indirectly dated to the 14th to 16th century CE (*34*), together with the published Tanzania_Pemba_600BP individual, documents the dispersal of ancestry related to present-day Bantu speakers throughout eastern Africa (Fig. 3 and table S5). This individual likely also reflects a Bantu-speaking population in Uganda during a period of complex-state formation in association with cattle keeping and cereal cultivation (*34*).

### Direct evidence of genetic exchange between Bantu and pastoralist/foragers in southern Africa

New ancient genomes from Botswana (three ancient individuals from the Okavango Delta region of northwestern Botswana and one from southeastern Botswana) allowed us to extend investigation of the spread of food-producing populations into southern Africa. Positioning on the PCA suggests mostly ancestry related to present-day Bantu speakers in these individuals (Fig. 2), and our modeling shows that the dominant genetic ancestry component in all four Botswana individuals is related to BantuSA_Ovambo, the Bantu-speaking southern African Ovambo (Fig. 3). Given the geographic position of the individuals and the genetic position on the PCA, we suspected another genetic ancestry component related to southern African hunter-gatherers. We therefore tested both South_Africa_2000BP and South_Africa_1200BP in two-way models for all four individuals (Figs. 2 and 3 and table S6). This provided working models for all individuals, with 30 to 40% southern African hunter-gatherer ancestry for the three individuals from the Okavango Delta (Nqoma and Xaro) and around 10% for the individual from the eastern border of Botswana (Taukome).

While, for the Nqoma and Taukome individuals, both southern African sources fit the data, for the two Xaro individuals, only South_Africa_1200BP provides a working fit, while South_Africa_2000BP fails (table S6). While South_Africa_2000BP has unadmixed southern African hunter-gatherer ancestry, South_Africa_1200BP was shown to be admixed with eastern PN-related ancestry (*3*), a pattern present in most Khoisan groups today (*1*). The fact that only South_Africa_1200BP provides a fitting model for the two Xaro individuals therefore suggests PN-related ancestry in these individuals, and we argue that our findings point to the presence of the same ancestry in the third individual from the Okavango Delta (Nqoma), although the low coverage in that individual prevents us from testing this. We also assessed whether the ancient Botswana individuals have differential ancestry to present-day Khoisan groups and found that only Juhoan_North stands out in that it has less affinity to ancient Botswana individuals compared to Gui, Naro or Juhoan_South (see table S9). An assessment using different Bantu sources in our qpAdm modeling shows that among different proxies of ancestry related to present-day Bantu speakers, only BantuSA_Ovambo, a group of southwestern Bantu speaker from Namibia, provides working models, while Tswana and Kgalagdi, who are most populations of Botswana and among southeastern Bantu speakers today, failed in our statistical modeling (table S6).

We confirmed PN-related ancestry by fitting three-way models with the Pastoral Neolithic individual Tanzania_Luxmanda_3100BP and South_Africa_2000BP as additional sources on top of BantuSA_Ovambo. Botswana_Xaro_1400BP and Botswana_Nqoma_900BP show 14 to 22% ancestral contribution from the PN source. Consistently, uniparental markers in the two individuals from Xaro support mixed ancestry. The first individual (XAR001) has mitochondrial haplogroup L3e1a2 and Y chromosome haplogroup E1b1a1a1c1a, both common in Bantu-speaking populations (*35*, *36*). The second individual (XAR002) has Y haplogroup E1b1b1b2b, associated with most ancient eastern African pastoralists analyzed here and previously (fig. S9), and also found in present-day southern African pastoralists (*37*), while his maternal lineage (L0k1a2) is possibly of indigenous South African Khoisan origin (*36*).

We assessed which ancestry (related to Neolithic pastoralists or Bantu speakers) admixed first with the South African forager-related gene pool using linkage disequilibrium decay (fig. S7) and could show that eastern African pastoralist-related admixture generally predates admixture from ancestry related to Bantu speakers. This is consistent with previous models of South African population history based on modern African genomes (*1*) and with linguistic (*7*) and archaeological (*38*) hypotheses for eastern African herders becoming established in this region before the Iron Age. We emphasize that our data do not address where the mixture between eastern herders and southern hunter-gatherers occurred. However, the aDNA data clearly point to the presence of already admixed southern forager and eastern pastoralist ancestry in the Okavango Delta by the late first millennium CE (Fig. 3). The order of admixture events in Botswana is directly supported by the ancestry mix present in the Okavango Delta individuals from a Bantu-related source and a South_Africa_1200BP-related source. Conversely, if admixture between ancestors of Bantu-speaking and eastern African herder populations had occurred before input of southern hunter-gatherer ancestry in southern Africa, then these signatures would be apparent in other regions, but, so far, early arrivals of Bantu speakers in nearby Malawi do not carry this eastern African component (*3*). Rather, in the most parsimonious model, initial population mixture occurred between groups related to South_Africa_2000BP and eastern African pastoralists (with South_Africa_1200BP being a descendant of that initial mixture). Bantu speakers arriving in southern Africa then mixed with this population giving rise to the individuals from Xaro analyzed here. No present-day population sampled so far has the same ancestry mix as the two Xaro individuals (as visible from the PCA; Fig. 2). While further sampling may still reveal such a population in the future, so far, this suggests that this population was later replaced by unadmixed Bantu-speaking populations, as inhabit the region today.

The arrival of East-African pastoralist-related ancestry in Botswana and South Africa has been associated with the emergence of lactase persistence (LP) in these regions, as found in some Khoe-speaking people today, such as the Nama (*39*, *40*). We therefore investigated whether any of the known SNP alleles associated with LP are present in the ancient Botswana individuals or any of the other African individuals reported in this study. Among eight LP-related SNP positions that are present in our 1240K capture panel, we found no evidence for the presence of any of these LP-associated alleles (table S7). We also examined malaria resistance genes, which have been linked to the spread of Bantu speakers, and found derived alleles in XAR002 at SNPs rs2515904 and rs1050829 (table S7), where derived alleles are associated with a higher risk to malaria (*41*), coinciding with the admixture with ancestry related to Bantu speakers found in the genetic profile of this individual (table S6).

Genetic results mirror archaeological data indicating diversity in the emphasis on farming, herding, and foraging between sites and communities during the early Iron Age of Botswana (*42*, *43*). As in eastern Africa, it appears that specific trajectories of interaction and integration in particular regional and temporal settings influenced the diversity in subsistence strategies that was a hallmark of African history until recent centuries.

### Historical individuals from Congo document ancestry related to Bantu speakers in Central Africa

Our most recent ancient genomes come from the west of the DRC (Congo_Kindoki_230BP and Congo_NgongoMbata_220BP) and show unadmixed ancestry related to present-day Bantu speakers, similar to the individual from Munsa analyzed above, clustering tightly together in the PCA with the published individual Tanzania_Pemba_600BP and some present-day eastern and southern African Bantu speakers (Fig. 2). Grouping Congo_Kindoki_230BP and Congo_NgongoMbata_220BP as a single genetic group, we tested their genetic affinity to present-day Bantu-speaking populations and ancient genomes related to present-day Bantu speakers, including Munsa, via outgroup *f3* statistics. Our samples share highest genetic affinity with the ancient individuals Tanzania_Pemba_600BP and Kenya_IA_Deloraine, followed by BantuSA_Ovambo. We further found no other population that has more genetic affinity to either the ancient Congo individuals or BantuSA_Ovambo than Tanzania_Pemba_600BP, using the symmetry test *f4* (Congo_Kindoki_NgongoMbata, BantuSA_Ovambo; X, chimpanzee) (fig. S6), which is also confirmed by qpWave (table S5). The fact that the ancient individuals with ancestry related to Bantu speakers are more closely related to each other than to present-day Bantu-speaking groups, despite the notable temporal and spatial distance between them, might reflect input of additional ancestral components in most present-day Bantu-speaking populations as a result of later migrations but could also be confounded by batch effects among aDNA samples being generally slightly attracted to each other compared to present-day genotyping data. It should also be noted that evident gaps in the sampling of present-day populations exist, including in the DRC itself and many neighboring countries.

The other ancient individual from Kindoki, Congo_Kindoki_150BP, presents a genetic makeup different from Congo_Kindoki_230BP, based on PCA and admixture analysis (Fig. 2). Again, grouping Congo_Kindoki_230BP with Congo_NgongoMbata_220BP, we performed *f4* statistics for testing whether Congo_Kindoki_150BP and the two other historic groups are genetically similar. As shown (fig. S6D), several west Eurasian groups (or ancient African groups carrying west Eurasian ancestry) are genetically significantly closer to Congo_Kindoki_150BP than to the other Congo individuals. When modeling Congo_Kindoki_150BP with qpAdm (Fig. 3B and table S5), we found a fitting model with 85 ± 7% ancestry related to Bantu speakers and 15 ± 7% ancestry related to western Eurasians. This ancestry profile would be consistent with the hypothesis that this individual has Portuguese ancestry, which would fit with the colonial history of the region (*44*) and the Christian burial of this and other individuals in Kindoki (see Supplementary Text).

## DISCUSSION

Our study documents the coexistence, mobility, interaction, and admixture of diverse human groups throughout sub-Saharan Africa over the past few thousand years by describing 20 new ancient genomes from Kenya, Uganda, the DRC, and Botswana. Together with previously published ancient African genomes (*3*, *4*, *9*), it demonstrates that, across all regions studied, the earliest visible ancestry is closely related to that of present-day hunter-gatherer populations such as the San in southern Africa, the Hadza in eastern Africa, and the Mbuti of the central African rainforest. Current data show that while this geographically defined forager population structure extends back to at least the mid-Holocene in eastern Africa (as represented by the 4500-BP individual from Mota), current forager populations reflect a contraction of ancestries that were once more spatially overlapping [as noted in (*3*) for eastern and southern hunter-gatherers]. Restriction of gene flow between regional forager groups in eastern, southern, and central Africa, whether over the long term due to climatic and environmental factors such as increasing aridity or later as a result of encapsulation by food-producing groups, has likely contributed significantly to the population structure observed in the African continent.

It is worth noting that, in some cases, overlapping forager ancestries could also reflect prefood-producing era migrations. For example, it is possible that the expansion of bone harpoon technologies (*45*), wavy-line pottery (*46*), and aquatic resource-based economies from northern to eastern Africa in the early Holocene also involved population migrations (*21*). The wetter climate conditions at the time may also have encouraged previously invisible east-to-west connections between hunter-gatherers in the central African rainforests and the eastern African Great Lakes, perhaps reflected in the Mbuti-related ancestry in our early sample from Kakapel.

Our six new individuals from the Pastoral Neolithic in Kenya were added to previous findings (*4*), demonstrating greater complexity in their ancestry profiles than previously observed for Pastoral Neolithic individuals from the same region (*4*). While this may be the result of population structure preventing random mating and homogenization, another explanation for this pattern is that early herders migrated south along multiple contemporaneous, but geographically distinct, routes in a manner similar to historic branching migrations of Maa, Ateker, and Surmic peoples across eastern Africa. In such a scenario, a single-base population in northern Africa may have branched into many as some herding groups moved along the Nile corridor, some through southern Ethiopia, and possibly some through eastern Uganda. Following varying trajectories, groups would have encountered different populations and formed diverse patterns of intercommunity relationships, resulting in more variable integration of ancestries. This model may help explain why stark variations in material culture, settlement strategies, and burial traditions are maintained for so long among PN populations with closely shared ancestries. Furthermore, detection of substantial eastern African forager ancestries late in the PN at Molo Cave indicates a longer persistence of indigenous foragers than is evident in the archaeological record (*25*). Despite appearing genetically homogenous overall, forager groups interacted with incoming herders with different degrees of resistance or integration (*27*) that affected the timing and structure of genetic admixture. Additional archaeological and archaeogenetic data are still needed to test this model and better reconstruct historically contingent patterns of migration and interaction.

Moving into the Iron Age, we again see evidence for multiple pathways of population movement in eastern, central, and southern Africa. The two Iron Age individuals from the Kakapel site near Lake Victoria document a more extreme (and near-complete) increase in Nilotic-related ancestry, possibly related to the arrival of the Luo, than the five previously published Iron Age individuals from the Central Rift Valley (*4*). The only explanation for this is that genetic turnover must have been region-specific and could have involved multiple divergent migrations. Our observation of PN-related ancestry in eastern Congo in the late Iron Age, as well as the lack of ancestry related to Bantu speakers there at that time, is, so far, an isolated find that calls for further investigations about the spread of PN-related ancestry in the west of the eastern African core region.

The interplay between incoming Bantu speakers (as evidenced by ancestry in present-day groups such as the Luhya and Kikuyu) and Iron Age Dinka-related ancestry remains unclear, including the question of whether farming spread exclusively through the expansion of Bantu-speaking populations, or also through local adoption (*47*). However, new ancient genomic data from this study track the footprint of migrating Bantu speakers further into the south. Our data document the arrival of people with ancestry related to Bantu speakers in Botswana in the first millennium CE and their admixture there with eastern African pastoralist and southern African forager ancestry. It provides evidence for interactions between three distinct lineages in the region, in line with the hypothesized arrival of Bantu-speaking communities into southern Africa by 1700 BP (*48*), and offers genetic support to the hypothesis of a pre-Bantu expansion of pastoralists into southern Africa (*3*, *7*, *38*).

Beyond the signature of ancestry related to Bantu speakers in southern Africa, we also find this ancestry in unadmixed form in historical individuals from Uganda and western Congo, which show a genetic profile similar to that of previously published individuals from Tanzania [Tanzania_Pemba_600BP (*3*)] and Deloraine Farm [Kenya_IA_Deloraine (*4*)], as well as present-day Southern Bantu speakers (BantuSA_Ovambo), consistent with the well-documented genetic homogenization caused by the Bantu expansion (*49*). Nonetheless, aDNA studies are beginning to reveal highly variable patterns of Bantu admixture with regional forager and pastoralist populations in sub-Saharan Africa, with unadmixed ancestry related to Bantu speakers persisting in the western Congo and Tanzania until the historical era, but evidence for noticeable admixture within centuries of initial arrival of Bantu speakers in southern Africa (*3*).

Our study highlights that while supraregional studies such as this one are important to understand continental-scale processes, increasingly regional-focused studies are called for in the future to better understand region-specific patterns of cultural and population changes (*4*). Important focal regions for these studies would include Sudan and the Horn of Africa to better understand the processes that brought the first herders into eastern Africa and regions to the north of Botswana, such as Zambia, to reveal more details about the interactions between early pastoralists and South African hunter-gatherers, as revealed by our individuals from Botswana. These studies are becoming more and more possible given the promising and increasing success rate of aDNA from Africa in a diversity of settings and time periods.

## MATERIALS AND METHODS

### Material collection

All sampling material from Kenya was sampled and exported under permits issued by the National Museums of Kenya and permissions from the National Commission for Science, Technology, and Innovation, Kenya. Material from Uganda was exported under a Ugandan government permit. Material from the DRC was excavated, sampled, and exported as part of the KongoKing project as outlined in text S1. The material from Botswana was exported under available permits from the Botswana government.

### Direct accelerator mass spectrometry ^14^C bone dates

We report 15 new direct accelerator mass spectrometry (AMS) ^14^C bone dates in this study from two radiocarbon laboratories (Oxford, 13; Glasgow, 3). Bone samples were prepared following the laboratory-specific protocol for radiocarbon dating. All ^14^C ages were calibrated with the IntCal13 Northern Hemisphere calibration curve (*50*) using OxCal version 4.3.2 (*51*). All uncalibrated, calibrated, and context-based dates are summarized in Table 1.

### aDNA sample processing

Originally, we screened 56 skeletal samples for DNA preservation from seven collections from different institutions (table S2) in dedicated clean rooms at the Max Planck Institute for the Science of Human History in Jena, Germany. DNA extraction and library preparation were performed with previously published protocols (*52*), including partial uracil-DNA glycosylase treatment (*53*) to reduce the characteristic deamination error of aDNA fragment. After screening, we enriched for 1.2 million informative nuclear SNPs (1240K) by in-solution hybridization (*54*) for 20 samples with ≥0.1% endogenous content. We processed DNA sequences using the EAGER v1.92.50 pipeline (*55*), with adaptors removed by AdapterRemoval v2 (*56*), reads mapped to hs37d5 by BWA alignment software v0.7.12 (*57*), and polymerase chain reaction duplicates removed by Dedup software v0.12.2 (*55*). We trimmed the first and last 3 base pairs (bp) of each read using *trimBam* function in bamUtils v1.0.13 (*58*). We applied a minimum base quality (Phred-scaled) of 30 and a minimum mapping quality (Phred-scaled) of 30- to 3-bp masked BAM files for contamination estimates and calling genotypes. We called a random allele for each target SNP after quality-filtering to produce a pseudodiploid genotype. For most of the downstream population genetic analyses, we used all autosomal SNPs from 1240K capture, while for a subset of analyses, we used transversions only to avoid the aDNA deamination error at transition sites. Mitochondrial DNA contamination was estimated using Schmutzi (*15*). For males, we estimated nuclear contamination using ANGSD v0.910 (*17*). All contamination estimates can be found in table S1.

### Uniparental haplogroup and kinship analysis

For mitochondrial DNA haplogroups, we used HaploGrep2 (*59*) and HaploFind (*60*) with mitochondrial consensus sequences generated by Geneious v10.0.9 (*61*) restricting to reads with a mapping quality of >30. The Y haplogroup was determined by yHaplo program (*62*). For each male individual, we used a pileup of 13,508 International Society of Genetic Genealogy (ISOGG) SNPs (strand-ambiguous ones were excluded) and randomly drew a single base representing the genotype at each SNP position, with the same quality filtering applied to genotyping autosomes. For the genetic relatedness, we calculated pairwise mismatch rates of pseudodiploid genotypes across all SNPs. In addition, we applied the software READ (*63*), which confirmed the kinship estimates from the pairwise mismatch rate. This analysis revealed that the two petrous bones from samples LUK001 and LUK002 are from the same individual, and we merged the two libraries.

### Present-day human data and published ancient genomes

We merged our newly reported ancient genomes published ancient African genomes (*2*–*4*, *9*–*11*), together with 584 individuals from 59 modern African populations (*1*, *12*, *64*, *65*) genotyped on the Affymetrix Human Origins array (Human Origins), and high-coverage genomes from the Simons Genome Diversity Project (*13*, *14*), including 300 individuals from 142 worldwide populations and 44 individuals from 22 African indigenous populations. Intersecting with SNPs present in the Human Origins array, we obtain data for 593,124 autosomal SNPs across worldwide populations.

### PCA and admixture-clustering analyses

We used smartpca v16000 from the EIGENSOFT v7.2.1 package (*66*) for PCA using all autosome SNPs and projected ancient individuals on eigenvectors computed from present-day African populations on the Affymetric Human Origin array with option “lsqproject: YES” on. We used ADMIXTURE v1.3.0 (*18*) for unsupervised genetic clustering analysis of ancient African samples along with present-day Africans and all published ancient Africans and Levant Neolithic individuals. One individual, NYA003, was removed from ADMIXTURE analysis due to its second-degree relationship with NYA002.

### Outgroup *f3* tests and symmetry *f4* tests

We performed outgroup *f3* with chimpanzee as outgroup, to check how our samples are closely related to present-day Africans and West Eurasians. The *f3* and *f4* statistics were calculated using the qp3Pop (v400) and qpDstat (v711) programs in the AdmixTools v5.1 package (*64*). We also performed model-based *f4* statistics for testing an additional genetic component in ancient eastern African Pastoralist groups (fig. S3). We used an in-house script that was first applied in (*10*) to compute *f4* statistics in form of (outgroup, test additional source group; two-way admixture model, Target). We used Ethiopia_4500BP + Levant_ChL as the hypothesized two-way admixture model and Dinka as the test additional source group and calculated model-based *f4* with varying Ethiopia_4500BP–related proportion in the two-way model from 0 to 100% in increments of 0.1%, with SE added by 5-centimorgan block jackknife method.

### qpWave and qpAdm analyses

For modeling ancestral components, we used qpWave v410 and qpAdm v810 (*65*) in the AdmixTools v5.1 package (*64*). Here, we used transversions only to avoid the artifact from aDNA fragments, and with “allsnps: YES” option on to maximize the allele frequency–based resolution. We use a set of 12 worldwide populations—Mbuti, Mende, Dinka, Khomani, Anatolia_Neolithic, Iran_Ganj_Dareh_Neolithic_published, French, Sardinian, Punjabi, Ami, Onge, and Karitiana—as outgroup in our test and move a certain population from the outgroup list into the reference population list if needed.

### Dating admixture

We used DATES v600 (*67*) for dating individual- and group-based admixture. A default bin size of 0.001 Morgans is applied in our estimates (flag “binsize: 0.001” added).

### Phenotypic SNP analyses

We examined SNPs encoding for biological traits in the newly reported ancient African genomes, such as LP, Malaria resistance, and eye/skin pigmentation, following the list of SNPs used in (*68*). For each phenotype-associated locus, we report the number of reads with derived alleles versus the total number of reads covered on this site in table S7, by applying SAMtools pileup on BAM files after quality filtering (*-q 30 -Q 30*).

## Supplementary Material

aaz0183_Tables_S1_to_S10.xlsx

aaz0183_SM.pdf

## SUPPLEMENTARY MATERIALS

Supplementary material for this article is available at http://advances.sciencemag.org/cgi/content/full/6/24/eaaz0183/DC1
