# Supplementary material for: Ancient genomes reveal complex patterns of population movement, interaction, and replacement in sub-Saharan Africa
Source: Sci Adv. 2020 Jun 12;6(24):eaaz0183. doi: 10.1126/sciadv.aaz0183 (PMC7292641; doi:10.1126/sciadv.aaz0183)
Supplement: aaz0183_SM.pdf [file aaz0183_SM.pdf]

Science Advances
AAAS

advances.sciencemag.org/cgi/content/full/6/24/eaaz0183/DC1

Supplementary Materials for

Ancient genomes reveal complex patterns of population movement, interaction, and replacement in sub-Saharan Africa

Ke Wang, Steven Goldstein, Madeleine Bleasdale, Bernard Clist, Koen Bostoen, Paul Bakwa-Lufu, Laura T. Buck, Alison Crowther, Alioune Dème, Roderick J. McIntosh, Julio Mercader, Christine Ogola, Robert C. Power, Elizabeth Sawchuk, Peter Robertshaw, Edwin N. Wilmsen, Michael Petraglia, Emmanuel Ndiema, Fredrick K. Manthi, Johannes Krause, Patrick Roberts, Nicole Boivin*, Stephan Schiffels*

*Corresponding author. Email: boivin@shh.mpg.de (N.B.); schiffels@shh.mpg.de (S.S.)

Published 12 June 2020, *Sci. Adv.* **6**, eaaz0183 (2020)
DOI: 10.1126/sciadv.aaz0183

The PDF file includes:

Supplementary Text
Figs. S1 to S9
References

Other Supplementary Material for this manuscript includes the following:

(available at advances.sciencemag.org/cgi/content/full/6/24/eaaz0183/DC1)

Tables S1 to S10

Supplementary Materials

Supplementary Text

Text S1. Ethical statement

This is an ethical statement regarding ancient individuals from Kindoki and Ngongo Mbata site in the Democratic Republic of the Congo (DRC). The KongoKing research project was conducted in the DRC authorized by the "Arrêté ministériel n°0115/CAB/MIN/JSCA/2012" dated August 8, 2012. Each year a separate document was signed between the KongoKing project mission Director and the Minister for Culture to authorize exportation for scientific study of artifacts and ecofacts. Furthermore, at the local village level, all surveys and excavations had to be accepted by the local community, often paying out a "droit de passage" to the land owner(s). Each time an ancient burial was encountered during the fieldwork, further work could be carried out only by organizing with the local specialized man the ritual to appease the dead spirit(s). At the end of any burial excavation, before filling up, another ceremony was conducted to thank the cooperation of the deceased person leading to not having had any incident.

Text S2. Archaeological information on newly reported individuals

Here we describe the archaeological sites at which our ancient individuals were found.

Lukenya Hill, GvJm 202, Kenya

Lukenya Hill is an Archaean Basement gneiss inselberg located in southern Kenya (−1.465°, 37.067°), in the Athi-Kapiti Plains east of the Central Rift Valley and just outside the current extent of Nairobi. The Lukenya inselberg has several dozen excavated archaeological sites including rockshelters with Middle and Later Stone Age components (*69*) as well as open-air Pastoral Neolithic sites (*70*, *71*) as well as rock-art occurrences and remains of historic to recent Maasai meat-feasting rituals.

Several major Neolithic sites are located at elevations of between 1600-1700m on Lukenya Hill, including GvJm44 which yielded charcoal radiocarbon dates of 3290±145, (GX5348) (*71*). Along with finds of "Nderit" pottery styles better known from 5000-4000 BP in Lake Turkana of northern Kenya, Lukenya Hill has been considered a major loci for the earliest occurrences of the Pastoral Neolithic in southern Kenya (*25*). An additional major Pastoral Neolithic (PN) site is GvJm184, which yielded slightly later dates of 2716-1735 cal BP (*71*, *72*). GvJm184 has yielded typical Savanna Pastoral Neolithic (SPN) pottery styles as well as remains of domesticated cattle, sheep and goat.

The site of GvJm202 is a rockshelter site including several human burials located nearby to the PN site of GvJm184, and so the burials were believed to be attributed to the Pastoral

Neolithic phase (*72*). Excavations found remains of at least 6 individuals consisting of 5 adults and 1 sub-adult (*73*, *74*). A sample from Skeleton C (labeled in original excavations) and two samples of loose human remains from Context 11 and Context 1 were sampled for aDNA. Samples from Skeleton C and the loose remains yielded aDNA sequences.

The loose remains were labeled in this study "LUK001" and the Skeleton C sample labeled in this study "LUK003" with dates of 3610–3460 cal BP and 3635–3475 cal BP., respectively. Given that these were very temporally close date ranges with similar genetic composition, both individuals are given the population label "Kenya_LukenyaHill_3500BP". LUK001 was found to be a male and LUK003 was found to be female (*contra* osteological indicators that were ambiguous-to-slightly-male, see (*73*, *74*)). With the exception of two individuals from PretteJohns Gully individual dated to ~4200 BP and presumed to be associated with an early movement of herders (*4*), the Lukenya individuals here represent the oldest examples of the migration event(s) responsible for the more pronounced expansion of herding detected all across Kenya and Tanzania (*3*, *4*).

Hyrax Hill, GrJj25, Kenya

Hyrax Hill is a major multi-component archaeological site and National Monument on the northeast shore of Lake Nakuru, Central Rift Valley, southern Kenya. Hyrax Hill has three major locales, of which two (Hyrax Hill I and Hyrax Hill II) have seen major excavations. Based on archaeological materials and pottery styles, primary deposits at the site belong to the Savanna Pastoral Neolithic (SPN) and Iron Age phases (*75*). Excavations by Mary Leakey from 1937-1938 revealed Neolithic and Iron Age village deposits which included hut and pit structures. The Neolithic component of the site included one of the largest formal cemeteries known for the Late Holocene of southern Kenya, containing over 18 burials within a central mound. Burials were shallow but otherwise variable, sometimes containing secondary burials and sometimes with multiple individuals apparently in a single pit. Large flat stones were placed over burial pits with stone circles resembling very small cairn-type features (*75*). According to the original report, nine individuals thought to be women were buried with large grinding stones or "platters".

Capping burials with shallow mounds and large stone slabs, and the heterogeneous style of burials, bear close similarities to the earlier Pastoral Neolithic traditions documented from 5000-4000 BP around Lake Turkana (*76*). Recovery of sedge beads and pestles, and obsidian blades associated with the burials is also comparable to the burial goods at the mortuary site of Njoro River Cave dated to c. 3100 BP around 20km west of Hyrax Hill. These two packages of mortuary characteristics are associated with the Nderit and Elmenteitan pastoralist traditions respectively, showing considerable overlap and interrelation between these archaeological entities that is now known from genetic results (*4*). SPN contexts at Hyrax Hill had not previously been radiocarbon dated, and common speculation was that site dated to around 2000 BP (*73*, *74*). A second burial area at Hyrax Hill consists of a double burial in a stone cairn associated with pit features thought to be Iron Age cattle enclosures. Additional excavations have taken place at the site, but have not yet been published.

The individual samples for this aDNA study all come from the SPN burial grounds excavated by Mary Leakey. The only individual to yield high aDNA coverage was found to be male and

was directly dated to 2365-2305 calBP, confirming estimates for the age of the site and its cultural attribution as Pastoral Neolithic.

Molo Cave, GoJi3, Kenya

Molo Cave is an archaeological occurrence in the Mau Escarpment around 50km west of Lake Nakuru in the Central Rift Valley of southern Kenya. The remains of three individuals were salvaged by Mary D. Leakey and deposited in the National Museums of Kenya, but there is very little archaeological or accession data to speculate on its cultural affiliation or context (*77*). It has been believed to likely date to the Pastoral Neolithic period (*73*). aDNA was recovered from two of the three individuals from this site, yielding dates of 1415-1320 calBP (MOL001) and 2110-1990 calBP (MOL003), confirming a likely Pastoral Neolithic attribution. The genetic composition of MOL003 strongly associates it with other Pastoral Neolithic samples, and MOL001 is somewhat intermediate between PN and Pastoral Iron Age samples. Coupled with its late date, it is possible MOL001 reflects admixture between PN and PIA populations at this later time.

Nyarindi Rockshelter, GqJc13, Kenya

Nyarindi is a rockshelter site along the southeast of Lake Victoria, Nyanza, Kenya. The site was informally excavated between 1939 and 1941 by a British deacon (surname Owen) (*78*). Deacon Owen did appear to record stratigraphic levels to some degree, but reported only on the early “Smithfield” levels at the lowest part of the archaeological sequence, which seem to be an Early Stone Age component. The upper layers are poorly recorded, however Owen recovered the remains of possibly five individuals, mostly in the form of mandibles and cranial fragments, with only a few post cranial fragments. The only artifacts from the upper portions of the excavations are a few pottery sherds and quartz stone tools. The quartz tools are non-descript but likely derive from Later Stone Age industries, and the pottery is typical Late Iron Age roulette decorated styles.

Two of the individuals (labeled here NYA002 and NYA003) of the three sampled by this study yielded genomic data. These were found to be a female and male respectively. A direct date on NYA002 came out to 3555-3375 calBP. Given this date and the genetic affiliation with other eastern African hunter-gatherer samples in this study and previously reported, it is most likely this individual is related to the Kansyore fisher-forager traditions of Lake Victoria. Pottery collected from the site must therefore post-date the burials by over 2000 years. As is expected, these individuals cluster with other hunter-gatherer samples from around Lake Victoria, including the Kenya_Kakapel_3900BP sample (*4*).

Kakapel, Kenya

The Kakapel (also called Kakapeli) Rockshelter site is a granitic tor within the Chelelemuk Hills south of Mount Elgon in North Teso, Busia County, of western Kenya. Iron Age rock art is visible within an overhang that is approximately 5m deep and 4-10m tall along the southern edge of the pluton. The area surrounding the rock art has extensive evidence for prehistoric occupation, and is where archaeological excavations have been focused. Kakapel is currently a protected site managed by the Trust for African Rock-Art (TARA) and the National Museums of Kenya and is surrounded by mixed-open forest and small patches

of pasture where cattle are occasionally left to graze. The entirety of the region surrounding the site is under agricultural cultivation, primarily for corn.

There was no record of excavations at the Kakapel Rockshelter site (except some sub-surface testing mentioned by Dr. Odak previously) until 2012 when a National Museums of Kenya(NMK) team initiated testing with a single 1x1 trench located near the rock art panel(Trench I).In 2015, the NMK returned for more extensive excavations including opening two new trenches (Trench II and Trench III). The NMK encountered human remains in both units (at ~35cm below surface in Trench II and ~1m below surface in Trench III). The individual (Burial 2) in Trench II was well preserved with almost all elements present, whereas the individual in Trench III (Burial 1) exhibited in-situ taphonomic crushing that destroyed much of the axial skeleton. Petrous bone from the Burial 1 individual, a tooth and right first metacarpal from the Burial 2 individual and one isolated tooth from a third individual found near Burial 2 were selected for aDNA sampling.

Kenya_Kakapel_3900BP

This individual (Burial 1) was in a primary, articulated burial in a shallow pit in Trench III. The body was flexed on its left side, oriented south-southeast to north-northwest and facing west. The skeleton was highly fragmentary and incomplete. Three iridescent perforated shell fragments were recovered near the face, with 25 disc beads made from various raw materials (including ostrich and land snail) were recovered near the legs. Contexts surrounding the burial contained pottery attributed to the Kansyore fisher-forager traditions around Lake Victoria.

Skeletal morphology suggests a male individual (confirmed by aDNA analyses) who likely died between 20-30 years old. The third molars had erupted and finished development, but were lightly worn. Dental health was otherwise good.This individual was directly dated to 3974–3831 cal. BP (3584±28 bp, SUERC-86057). These dates are consistent with associated material culture suggesting a connection with Kansyore fisher-forager traditions that existed around Lake Victoria through the Holocene. Isotopic analyses of tooth enamel from the individual yielded a delta 15N/14N of 7.76 and a delta 13C/12C of -15.7, far lower than the -4 to -8 values from fauna from the same levels. While the individual appears to have had a major protein component of the diet, he was either consuming other faunas that grazed on C3 plants or had a diet largely based in direct C3 plant consumption.

Kenya_Kakapel_300BP

This well-preserved individual (Burial 2) was in a primary burial in Trench II. The body was semi-flexed on their left side, oriented southeast-northwest with the head facing south-southwest.There was no evidence of a burial pit, although the remains were placed between large pieces of roof fall.This individual was directly dated to 309-145 cal. BP. Skeletal morphology is consistent with a female individual (confirmed by aDNA) who died in middle adulthood. Complete fusion of the sphenooccipital synchondrosis and medial clavicle indicate an age over 30, while age estimates from the pubic symphyses, pelvic auricular surfaces and first rib suggest an age range of mid 30s-50s (*79*, *80*). Stature estimates based on the complete right humerus, radius, ulna, femur, and fibula range between 153.5 ± 4.25cm and 158.0 ± 5.05cm, with a mean of 156 cm or about 5’1.5 (*81*).

Kenya_Kakapel_900BP
This individual is represented by an upper right lateral incisor found in the Burial 2 fill but that did not belong to that individual. The tooth was directly dated to 910-736 cal. BP. An upper right canine, potentially from the same individual, was recovered from a greater depth. While this individual cannot be attributed to any clear material culture, the radiocarbon date places it within the middle-to-late phases of the Iron Age, probably with the Roulette pottery traditions of the African Great Lakes region for this time.

Munsa, Uganda

Munsa is one of several sites with earthworks in western Uganda first reported in the colonial period (*82*). The earthworks comprise systems of ditches, commonly up to 4 meters deep and often encircling one or more hills and dated to approximately 500 calBP. The function of the earthworks is uncertain (*83*). Those at Munsa, which surround a flat-topped rocky hill, were first mapped by Lanning (*84*). Excavations at the center of the site in the 1990s (*34*) uncovered numerous grain-storage and other pits, some with burials, as well as an iron-smelting furnace (*85*), numerous potsherds, bones of cattle and other animals, and occasional iron artifacts, glass beads, and grinding stones. Many of these features and artifacts date to the centuries immediately prior to the construction of the earthworks, which are linked to oral traditions of an ancient kingdom (*86*, *87*).

Individual H.s.#7, analysed here, was recovered from Context 250 (a pit fill) in Unit 101E/84N at Munsa A, i.e. on top of the hill at the center of the earthworks. The skeleton was found in a bell-shaped pit that was likely used originally for grain storage. The articulated skeleton is that of a woman aged around 35 – 50, and our genetic analysis confirmed the female sex. The skeleton was laid out on a NW-SE axis, extended, lying on her back with the head looking left (north). There was an iron bangle associated with the body. The pit contained another female human skeleton, with a few associated glass beads, lower down that had been disturbed when H.s.#7 was interred. Also in the pit were charcoal, sherds, grindstone fragments, a few pieces of iron slag, and animal bones, as well as an almost whole pot and large quern at the same level as H.s.#7.

Not enough material for direct dating of H.s. #7 was available to us. An AMS date on charcoal from the same context as the lower burial (#13) resulted in a weighted average of 955 +/- 40 bp. There is also an AMS date on charcoal from a context above H.s.#7, probably around the very top of the fill of the pit of 485 +/- 45 bp . Collagen from H.s. #13 yielded an AMS date of 345 +/- 45 bp, although based on low amounts of collagen. This collagen date of 345 bp is younger than that of the charcoal date from much higher in the pit fill of 485 bp, with a small overlap at the sigma-2 range the late 15th century AD. It is possible that charcoal was added to the pit fill that came from an older context elsewhere on the site. Given also dates from pits nearby, we estimate the date of H.s. #7 therefore to the 14th – 16th century AD, approximately contemporary with the construction of the earthworks.

Matangai Turu Northwest, the DRC

The rock shelter site of Matangai Turu Northwest is located at 800 m above sea level in the Ituri rainforest, the DRC (*88*). This 7 metre high granite shelter lies 95 m from the Andeilu river and was known to have been recently occupied by Efe foragers when it was excavated

in the late 1990s by Julio Mercader and colleagues (*88*). The team excavated a partial human skeleton during these excavations from Level 5, directly dated to the Late Holocene (813 ± 35 ^{14}C years BP; 1218-1277 AD calibrated age (1 sigma) (UtCNr 5074). The skeleton was associated with lithics identified as 'Late Stone Age' type, animal bone and shell remains from wild taxa, fruit endocarps from forest trees, and phytoliths from tropical forest plants. Phytolith analysis indicated that the habitat was dense tropical forest, without evidence of domesticated food(*89*). Overall, this individual was considered to be heavily reliance on foraging, while tooth health, modifications, as well as stature were used to tentatively suggest that this individual came from a population with a "pygmy" phenotype (*88*). Intriguingly, however, associated findings of Late Iron Age ceramics and an iron burial good hinted at the possibility of affiliation with Nilotic, central Sudanic farming populations (*88*, *90*).

Kindoki, the DRC

Kindoki was excavated between 2012 and 2014 under the direction of B. Clist as part of the KongoKing project (2012-2016, ERC Starting Grant n° 284126) led by K. Bostoen at Ghent University, Belgium. The site (-5.086°,15.129°) is located in the current-day Congo Central province of the DRC, 95km southwest of Kinshasa and 10km northwest of Kisantu (*91*, *92*). The excavations on the large hilltop – some 537 m^2 in total – were focused on the Late Iron Age and on historical domestic layers and pits related to the Congo kingdom. The Iron Age sequence with 16 ^{14}C dates starts with Kindoki ware whose producers settled on the hilltop from circa calAD 1300 to 1450 (*93*), i.e. before the arrival of the Portuguese at the Congo mouth on the Atlantic Coast in 1482. Slightly later, a continuous occupation extends from the late 15th century to the early 19th century. The higher number of artifacts during the 17th-19th centuries combined with the presence of a cemetery of 11 tombs suggests that Kindoki was only then the center of Mbanza Nsundi, the capital of the Kongo kingdom's Nsundi province. According to oral traditions, the Kongo kingdom would have been founded in the 13th century (*94*). In 1491, the Kongo king Nzinga a Nkuwu, was the first to convert to Christianity and to become João I of Kongo, followed a few weeks later by his son Mvemba a Nzinga, becoming Afonso. In 1495, Afonso was exiled to Mbanza Nsundi together with Portuguese missionaries. The town then turned into a centre of local Christianity. After his enthronement in 1509 as king Afonso I, he developed Christianity into a royal cult to which Kongo nobles adhered.

The Kindoki cemetery was excavated in 2012 (tombs 9 and 13) and 2013 (tombs 1-2, 4-8, 11-12) (*91*, *92*). It is interpreted as being the burial site for governors of the Nsundi province and their close relatives from the second half of the 17th century to the first half of the 19th century. The 11 tombs were constructed in close proximity to each other and oriented north-east/south-west at 220°, most of them in a similar way: a rectangular pit dug down to circa 2m, a rectangular surface demarcation of stones either lying flat or on their side, a topping pavement with more or less well-dressed stones, and a covering cairn of stones of various sizes and shapes on top of the rectangular structure. Where identifiable, the deceased were deposited on their back, in an extended position. According to the full skeletal analysis (*95*, *96*), parietal bones were only preserved in five tombs: 1, 7, 8, 9 and 11. DNA results for KIN003, KIN004 and KIN002 come respectively from tombs 9, 1 and 8.

Tomb 9, a 35-40 years old man of 1.61m estimated height (*95*, *96*), with 1 musket on his left side, 2 iron bracelets, 18 wound Venetian glass beads with floral inlays. Charcoal found on his right side was dated by Beta-333285: 190+/-30 bp, 1665-1950 calAD. Combination of artifacts and ^{14}C gives this more precise chronology: 1690-1725 (musket), 1725–1850 (beads), 1665–1817 (^{14}C; 66 % probability) (*91*, *97–99*). The new ^{14}C date on bone is OxA-37354: 172 ± 20 bp, 1672-1950 calAD (36% probability: 1672-1744; 40% probability: 1796-1895), confirming the former one on charcoal.

Tomb 1 contained a 30-35 years old man (*95*, *96*) buried without any associated artifact. Specific wear on several teeth points to a regular use of a smoking pipe, probably in clay. OxA-37355 is the first ^{14}C date obtained for this grave: 241 ± 20 bp, 1650–1799 calAD (67% probability: 1735-1799).

Tomb 8, an adult of *circa* 40 years old, osteologically determined as female with 1.57m estimated height (*95*, *96*) buried with 1,140 wound red-on-white glass beads, 14 wound pentagonal blue glass beads, 1 wound round white glass bead, 1 wound round blue glass bead, 3 internally silvered blown glass beads, 1 copper bead, 32 crotal bells, 660 *Pusula depauperata* sea shell beads, 1 *Tympanotonus fuscatus radula* mangrove shell, 1 iron anklet, 1 iron necklace, 1 copper chain, 1 gold chain, and large parts of a shroud (*91*, *97*, *99*). According to the glass beads types, the burial dates to 1825–1845. The new ^{14}C date is OxA-37353: 217 ± 20 bp, 1656–1805 calAD (76% probability: 1727-1805), which is considerably older.

While the molecular sex is male, several pieces of archaeological evidence are more typical for the burial of a female, first and foremost the measurements carried out on the mandible, the teeth and the long bones (*95*, *96*). Moreover, the funeral material of Kindoki graves 4, 5, 6, 7 and 12 containing swords (as well as four graves in Ngongo Mbata's church cemetery, see below) is notably different from that of Kindoki tombs 8 and 11 with hundreds of glass and shell beads, sometimes associated with thick iron anklets (see also several graves in two cemeteries at Ngongo Mbata and the Kulumbimbi church burial in Mbanza Kongo). The first type of tombs has commonly been identified as male, the second type as female in line with ethnographical studies from the 19th and early 20th century.

Ngongo Mbata, the DRC

Ngongo Mbata was excavated between 2012 and 2015 (total of 847,5 m^2) under the direction of B. Clist as part of the KongoKing project (2012-2016, ERC Starting Grant n° 284126) led by K. Bostoen at Ghent University, Belgium. The site (-05.806°, 015.124°) is located in the Kongo Central province of the DRC, 14 km north-east of Kimpangu and some 8 km from the Angolan border located to its south (*100*, *101*). The 18 ^{14}C dates obtained point towards human presence during the Late Stone Age and Late Iron Age, i.e. the hunter-gatherers around 9,000-8,000 bp and a settlement probably starting in the 16th century AD. According to the historical records available (i.e. chronicles and maps), Ngongo Mbata was the main and most affluent center of the Kongo kingdom's Mbata province in the 17th century. It developed to the second largest town in the kingdom because it was an important marketplace on a long-distance trade route connecting it to the kingdom's capital and the Atlantic coast. It was a centrally located thoroughfare in-between the ocean harbors in the west and the Kwango River valley in the east. At its apogee, Ngongo Mbata covered 50 hectares, at least. Along with Kongo people of different descents, it hosted Europeans of diverse origins (Portuguese, Spanish, German, Dutch), mostly merchants but also clergy. Ngongo Mbata is unique in that it had a stone church dated by excavations to the second quarter of the 17th century. Such stone churches were then only found in Mbanza Kongo, the Kongo kingdom's central capital in northern Angola and in the Portuguese territories south of it. In the DRC, the Ngongo Mbata church is the oldest surviving one.

Four cemeteries have been identified at Ngongo Mbata. Cemetery n°1, within the walls of the early 17th century stone church, was excavated in 1938 (*102*) and 2014 (*101*), and consists at least of 38 burials dating back to the 17th and 18th centuries. Cemetery n°2, located some 200 meters south-west of the church, contained at least four tombs, all very close one to the other. The only tomb studied is certainly younger than 1692 as evidenced by a 20 *reis* Portuguese coin minted between 1692 and 1699, which was found alongside three crucifixes. The tomb probably dates back to the early 18th century (*101*). Cemetery n°3, found to the south-west of the church on the southern side of a large plaza set up to the west of the church entrance, consisted of at least three tombs widely spaced, each marked by a pavement of large stones set over a tomb dug down to a depth of about 1.6m (*101*). Tomb 1 contained an adult male whose well-preserved skeleton was chosen for DNA identification. Cemetery n°4 was found while excavating large pits to the south of the church extending under a more recent house, probably a priest's residence (*101*). Three burials were identified, one set up in a refuse pit whose filling started after circa 1630 AD when the church was built.

Tomb 1, cemetery n°3, a man of circa 20 years of age and 1.7m estimated height (*95*). The rectangular pit was dug to 1.6m deep. The deceased lay on his back in an east-west position, as in all other burials studied at Ngongo Mbata, with two small glass beads near the neck (*99*, *101*). According to Christian customs of that time, the eastward orientation of his head suggests that he was a priest. After starting filling the burial, stone blocks were set up at a depth of 1,4m, i.e. on top of the body. Specific wear on several teeth points to the regular use of a smoking pipe, probably in clay. OxA-37363 is the first ^{14}C date obtained for this grave: 211 ± 21, 1657–1809 calAD (74% probability: 1724-1809).

Xaro, Botswana

Xaro is located on a former island silted to form a peninsular in the Okavango River Panhandle some 40km northwest of Nqoma. The site was excavated by Wilmsen and Denbow (1983) and subsequently by Wilmsen and Thebe. The pottery has distinctive single/multiple-row fingernail and stylus punctate motifs, multiple bands filled with incised or comb-stamped lines, as well as rhomboidal incised motifs; these motifs are identical to those found in the lower, Divuyu, levels of Nqoma securely dated to the 7th-8th centuries as well as at Ruuga some 230km upstream in Namibia firmly dated (by charcoal in sherds) to the 6th-7th centuries (*103*).

Not enough charcoal was recovered from Xaro to attempt reliable radiocarbon dating; however, seriation of Xaro pottery motifs confirms that the Divuyu-Ruuga date range also applies to Xaro. The site has been subject to periodic inundation which has destroyed all organic matter, including bones, so little can be said about subsistence. This inundation also contaminated charcoal to the extent that reliable radiocarbon dating is not possible, but the Divuyu levels at Nqoma are well dated to the 7th-8th centuries and Ruuga is securely dated by charcoal in sherds to the 6th-7th centuries. Seriation of the pottery confirms that this date range also applies to Xaro. Unlike at Nqoma, Xaro has no stone tools or other artifacts that would indicate an earlier hunter-gatherer component, and the site is considered to be related to early farmer occupations.

Two burials were excavated at Xaro located outside of the residential area of the site. Both were individual burials of adults found in flexed positions. As is typical for Iron Age burials, the individuals were interred with pottery, which matched the typical Early Iron Age styles found throughout the deposits at Xaro. The individual sampled for this analysis was an older

adult (likely 40-50 years old); the skull has morphological and craniometrical characteristics described as similar to both recent Khoisan and Bantu-speaking peoples of southern Africa.

Isotopic analyses of the Xaro individuals revealed very low $\delta^{13}C$ from samples of bone collagen (between -16.6 and -16.9) (*104*). The strong C3 values of the Xaro individuals compared to other Early Iron Age populations was interpreted as evidence for a substantial contribution of freshwater fish to the diet, which presents C3/C4 ratios similar to diets of C3 pathway plants (*104*). Given the sites location along the Okavango River, this hypothesis seems probably and the strong freshwater fish signature likely indicates that an aquatic carbon reservoir effect would impact the accuracy of any direct dates on human bone from the site.

Nqoma, Botswana

Nqoma is an open-air site on a low plateau of the Tsodilo Hills in northwestern Botswana some 50km west of the Okavango Delta. The Hills are composed of almost unlimited quantities of red specular haematite and sparkly micaceous schist; both minerals are highly valued as cosmetics for which hard rock adit mining was carried out on a massive scale, most intensively during the period AD 750-1025 (*105*). Nqoma was excavated by Denbow and Wilmsen from 1979-1980 and in 1985 with testing in several areas of the site covering roughly 100 by 200 sq. m (*106*, *107*).

Excavations yielded abundant archaeological remains, including stone tools, metal artifacts, animal bones, macrobotanical remains, Zhizo glass beads, and marine shells from the Indian Ocean trade (*106*). Much of the archaeology was concentrated in a stratified midden deposit that varied from .5 to 1 meter in thickness, below which sediment was found to be largely sterile. Radiocarbon dates on charcoal across the site revealed a consistent sequence from 1200-750 BP (*43*). Analysis of the faunal remains revealed that herding of sheep and goat was present by c. 1400 BP, with an increasing emphasis on cattle herding becoming apparent after 1150 BP (*43*). Wild fauna and large proportions of fish remains and freshwater molluscs were present throughout the sequence, indicating a strong reliance on wild resources. The riparian component may also reflect exchange with groups living along the nearby Okavango River and/or Delta. Excavations also yielded a diverse plant diet including wild marula and mongongo nuts, as well as domesticated sorghum and pearl millet (*104*, *106*).

Three human burials were excavated at Nqoma, all originating in the north-eastern section of the site. Human burials at the site include an infant interred between two parallel stone slabs, and an adjacent burial of a young woman with features that suggested affinities to Khoisan populations (*106*). This latter burial was also laid in a flexed position along the left site with the body oriented westward such that the head was directly facing the infant burial (*106*).

Below these burials was a previous burial of an older adult woman with skeletal features identified as “Bantu”, placed in an upright flexed position facing eastward (*106*). This individual from the earlier occupations of the Nqoma site was the one sampled for ancient DNA in this study. The burial included Nqoma style pottery. The burial had an intact elaborately decorated shallow ceramic bowl at her feet; this bowl was covered by two

segments from much larger vessels. A radiocarbon date of c. 980 BP was obtained from associated charcoal. While isotopic data indicated a diet including a major C4 component (*104*), the presence of fish remains throughout the Nqoma midden raises similar questions to Xaro regarding reservoir effects, and so the associated radiocarbon date for this burial pit and related material culture provide the most reliable age estimation.

Taukome, Botswana

Taukome Hill is a basalt inselberg some 700km crow-fly kilometers southeast of Nqoma in the hardveld of eastern Botswana excavated by Denbow in 1979 (*108*). Its most prominent feature is a large midden about 70m in diameter and 1.5m in depth indicating an extended occupation history initially confirmed by uncorrected radiocarbon readings and later calibrated to ca. AD 670-880 at the bottom and ca. AD 980-1150 at the top (c. 1240-995 BP).

This sequence encompasses the Zhizo (called Taukome, after this site, in Botswana) and Toutswe facies continuum of the larger Shashe-Limpopo region in which the site is a westernmost location near the Kalahari margin. Zhizo is a major division of the Nkope Branch of the Eastern Stream Urwere Tradition (*109*); thus, Taukome-Toutswe wares are markedly different from the Western Stream Kalundu wares at Nqoma indicating they were made by distinctly different Bantu-speaking peoples. A few Zhizo beads were found in the upper levels as were typically Zhizo clay human figurines. Cattle and sheep/goats were prominent in the economy, constituting 82% of the fauna based on MNI. The presence of grain bins at the site also indicate that agriculture was important (*108*).

Denbow excavated five burials including three adults and two juveniles within a kraal (livestock pen) feature at the site (*42*). The individual sampled here was an adult male, estimated to be around 40 years of age. Isotopic analyses of the Taukome burials demonstrated $\delta^{13}C$ values between -7.4 and -8.2, and $\delta^{15}N$ values between 9.5 and 10.3, largely consistent with the nearby Iron Age site of Kgaswe (*42*). Based on comparisons with isotopic signatures of fauna from these sites, it is likely that despite evidence for agriculture the isotopic signatures at Taukome can be explained through consumption of domesticated livestock (*42*).

Supplementary Figures

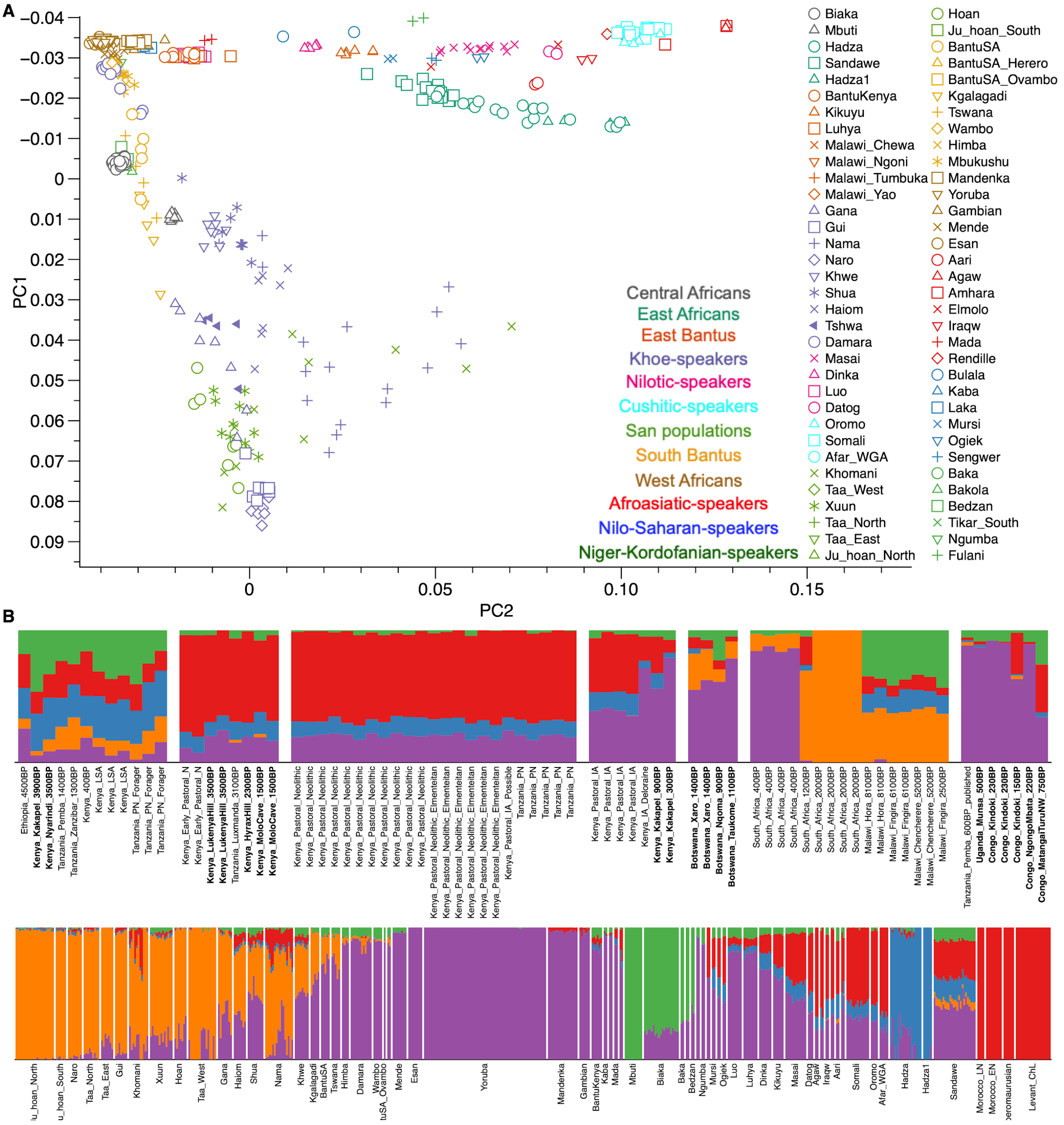

Figure S1. (A) PCA of present-day African and Levantine populations used in Fig. 2. (B) Admixture clustering analysis (K = 5) using all populations shown in PCA in Fig.2.

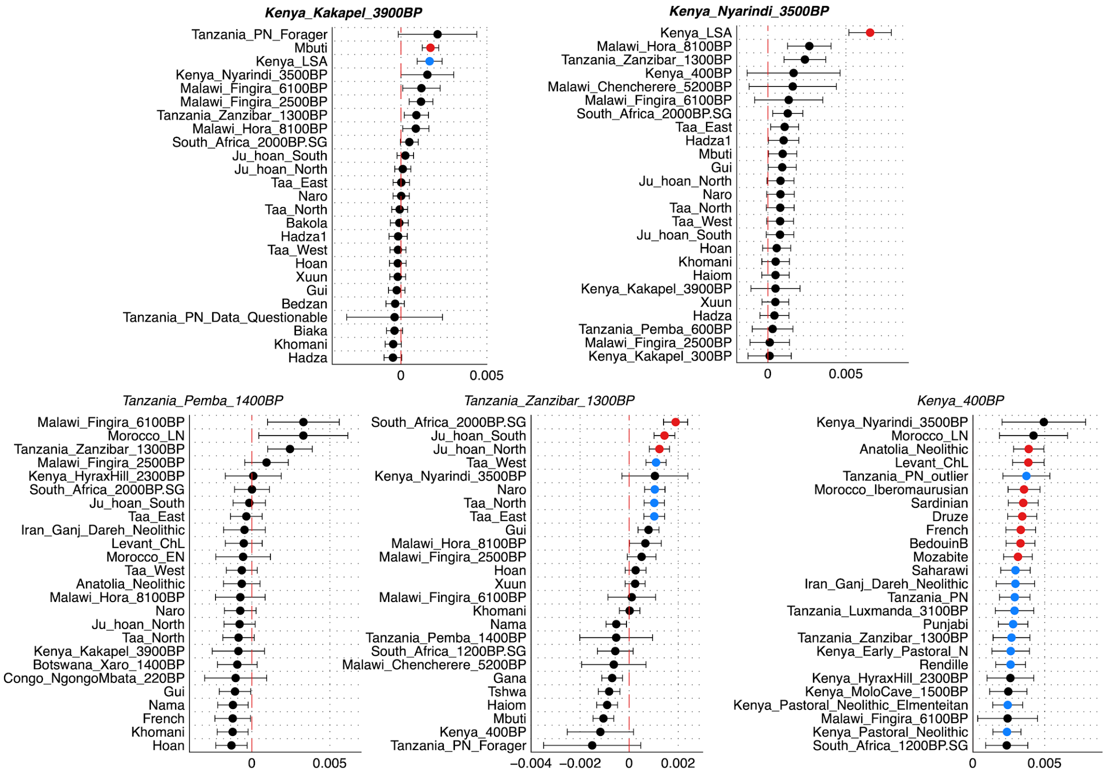

Figure S2. Testing the genetic affinity against Ethiopia_4500BP within the ancient eastern African forager cluster via *f4*-statstistics. For groups/individuals in the ancient eastern African forager cluster, we tested against all populations shown on PCA using symmetric *f4*(ancient east African forager, Ethiopia_4500BP; X, Chimp) and plot 25 groups with top *f4*-value**.** *f4-statistic* tests with Z-score ≥ 3 are shown in red, and tests with 2 ≤ Z-score < 3 are shown in blue.

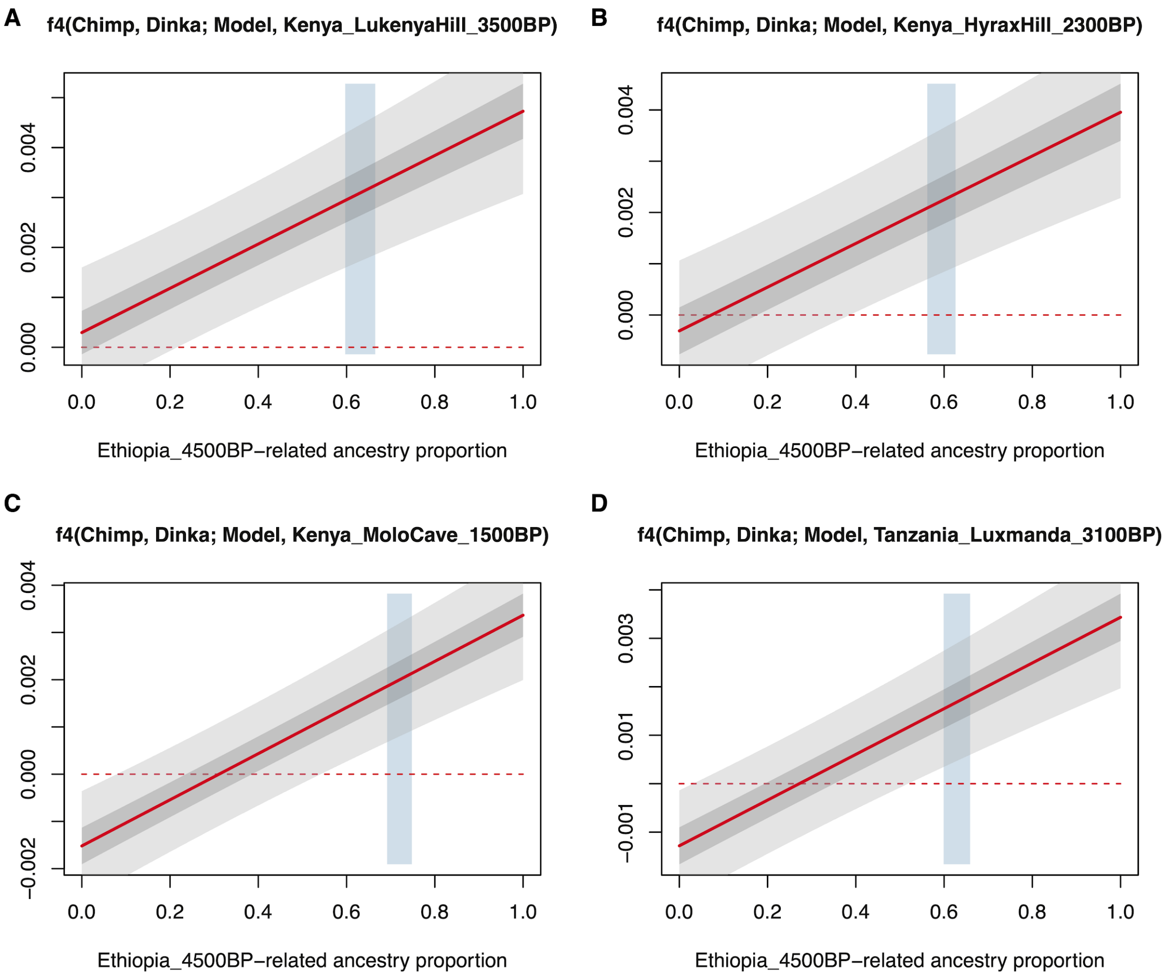

Figure S3. Testing additional genetic affinity to Dinka for ancient eastern African pastoralists based on a two-way admixture model of Ethiopia_4500BP and Levant_ChL . We calculated f4 statistics in form of (Chimpanzee, Dinka; Ethiopia_4500BP+Levant_ChL, ancient Eastern African Pastoralist) for (**A**) Kenya_LukenyaHill_3500BP, (**B**) Kenya_HyraxHill_2300BP, (**C**) Kenya_MoloCave_1500BP, (**D**) Tanzania_Luxmanda_3100BP. Positive F4 values suggests extra affinity to Dinka(above red dashed line), and negative F4 values suggests extra affinity with model. On x-axis, proportions of Ethiopia_4500BP-related ancestry range from 0 to 100% in increments of 0.1%. The blue shade marks the range of ancient Ethiopian ancestry proportion estimated by qpAdm (±1SE). Dark gray and light gray represent ±3 and ±1 SE ranges, respectively. SEs were calculated by 5-centimorgan block jackknife method.

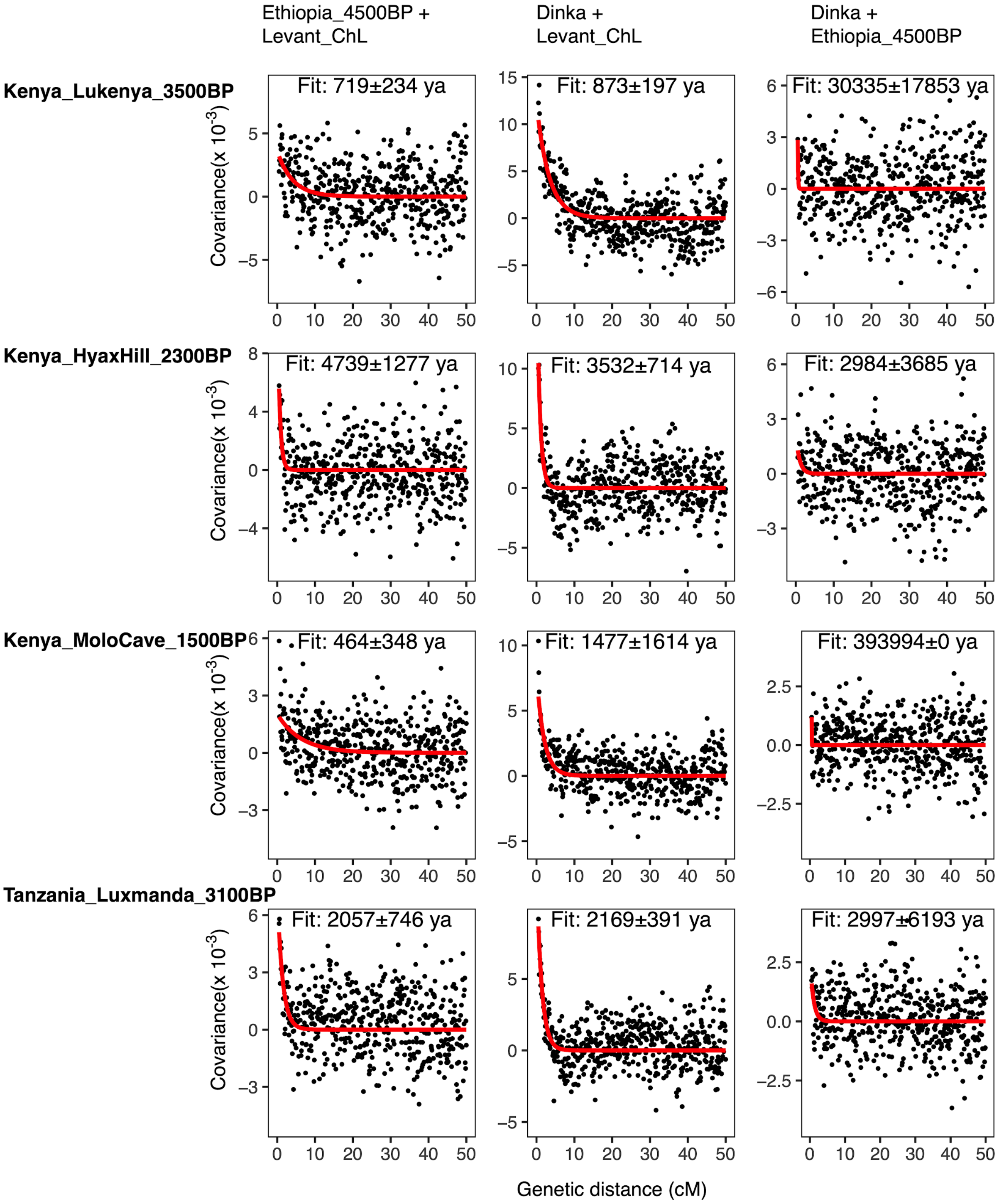

Figure S4. Dating the introduction of ancient Levantine and Dinka ancestry into ancient eastern African pastoralists using DATES. Fitted Linkage Disequilibrium decay curves are shown in red. Bin size of 0.001 Morgans is used for estimation in DATES. Same color and bin size is applied to Fig. S6. We dated three combinations of 2-way admixture from three sources: Ethiopia_4500BP, Levant_ChL, Dinka, shown in different columns, for four target pastoralist groups shown in different rows. DATES-inferred admixture dates between Levant_ChL and two African group show rather reasonable consistent estimates ranging from 2000-7000 years ago. While the admixture date estimates between Dinka and Ethiopia_4500BP are off scale.

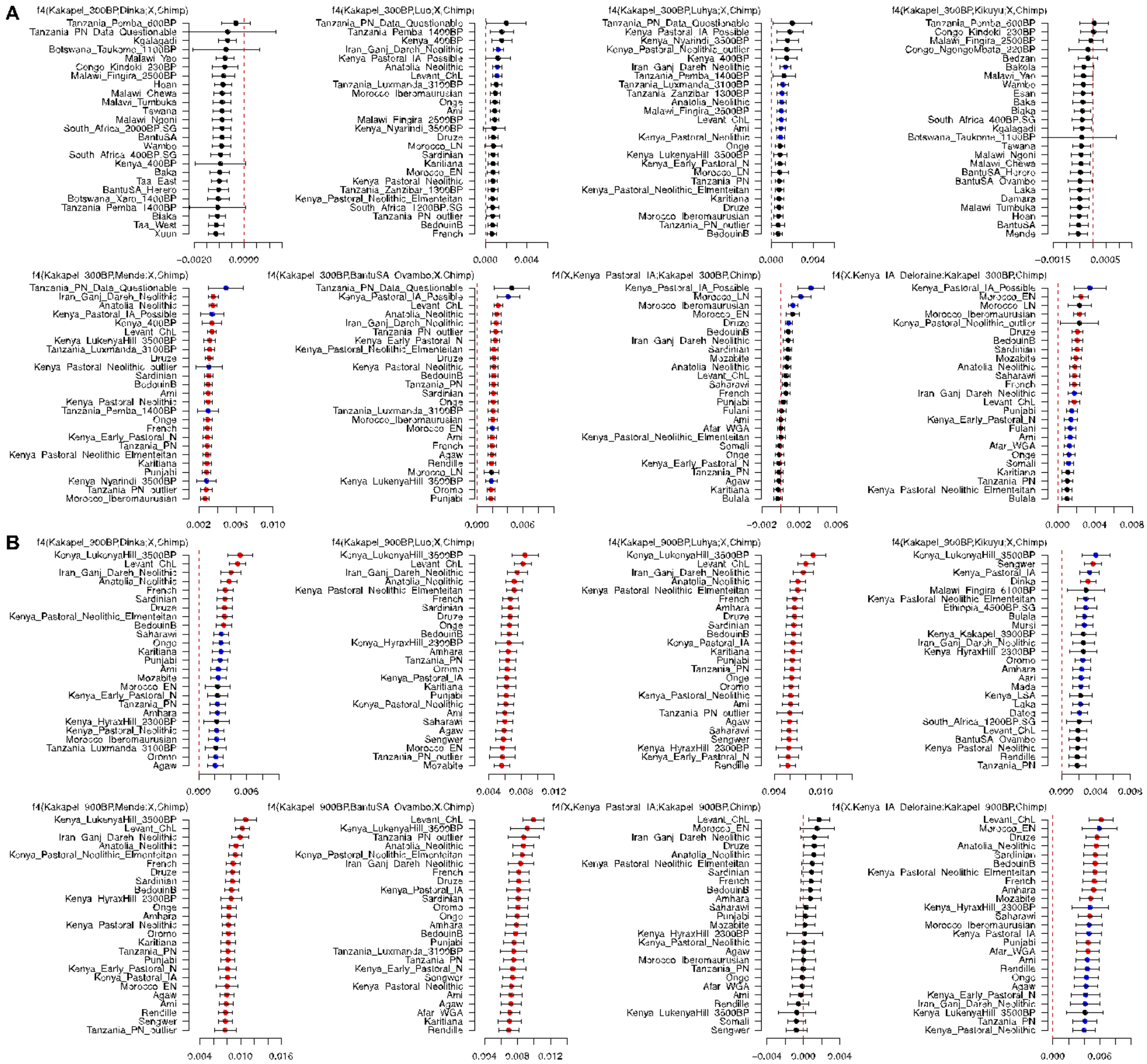

Figure S5. *f-statistics* for ancient individuals from Kakapel site, Kenya. (A) Comparing genetic affinity of Kakapel_300BP to present-day Nilotic-/Bantu-speaking populations and published ancient East African Iron Age genomes. **(B)** Comparing genetic affinity of Kakapel_900BP to present-day Nilotic-/Bantu-speaking populations and published ancient eastern African Iron Age genomes. *f4-statistic* tests with Z-score ≥ 3 are shown in red, and tests with 2 ≤ Z-score < 3 are shown in blue.

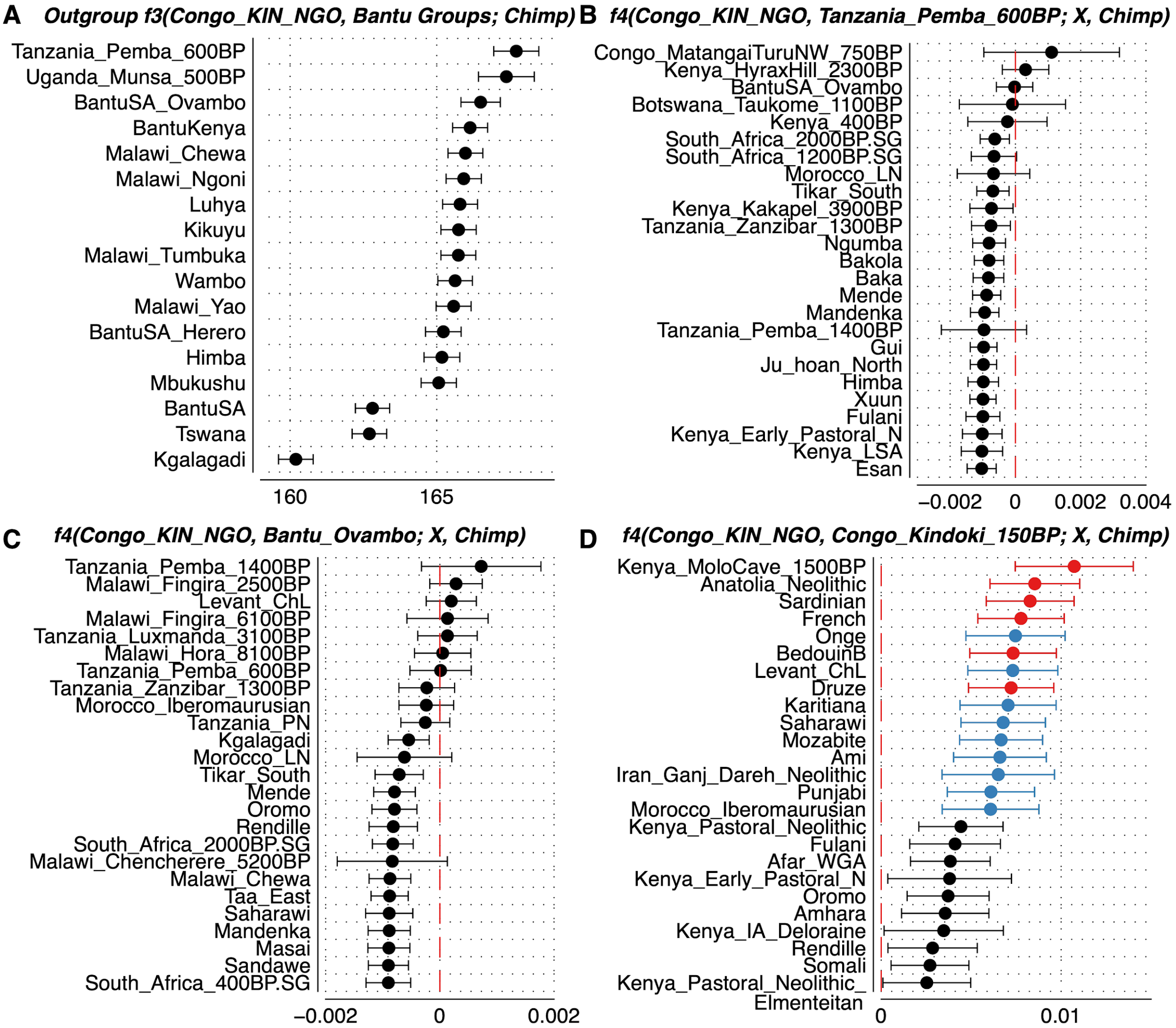

Figure S6. *f-statistics* for ancient individuals from the DR Congo. (A) Comparing the genetic affinity of historical individuals from the DR Congo to ancient Bantu-related groups and present-day Bantu-speaking groups.(B) Testing genetic symmetry between Tanzania_Pemba_600BP and main cluster of historical DR Congo genomes. (C) Testing genetic symmetry between Bantu_Ovambo and the main cluster of historical DR Congo genomes (merged as a single genetic group – "Congo_KIN_NGO"). (D) Cladility *f4* test between Congo_Kindoki_1500BP and the main cluster of historical DR Congo genomes. *f4-statistic* tests with Z-score ≥ 3 are shown in red, and tests with 2 ≤ Z-score < 3 are shown in blue.

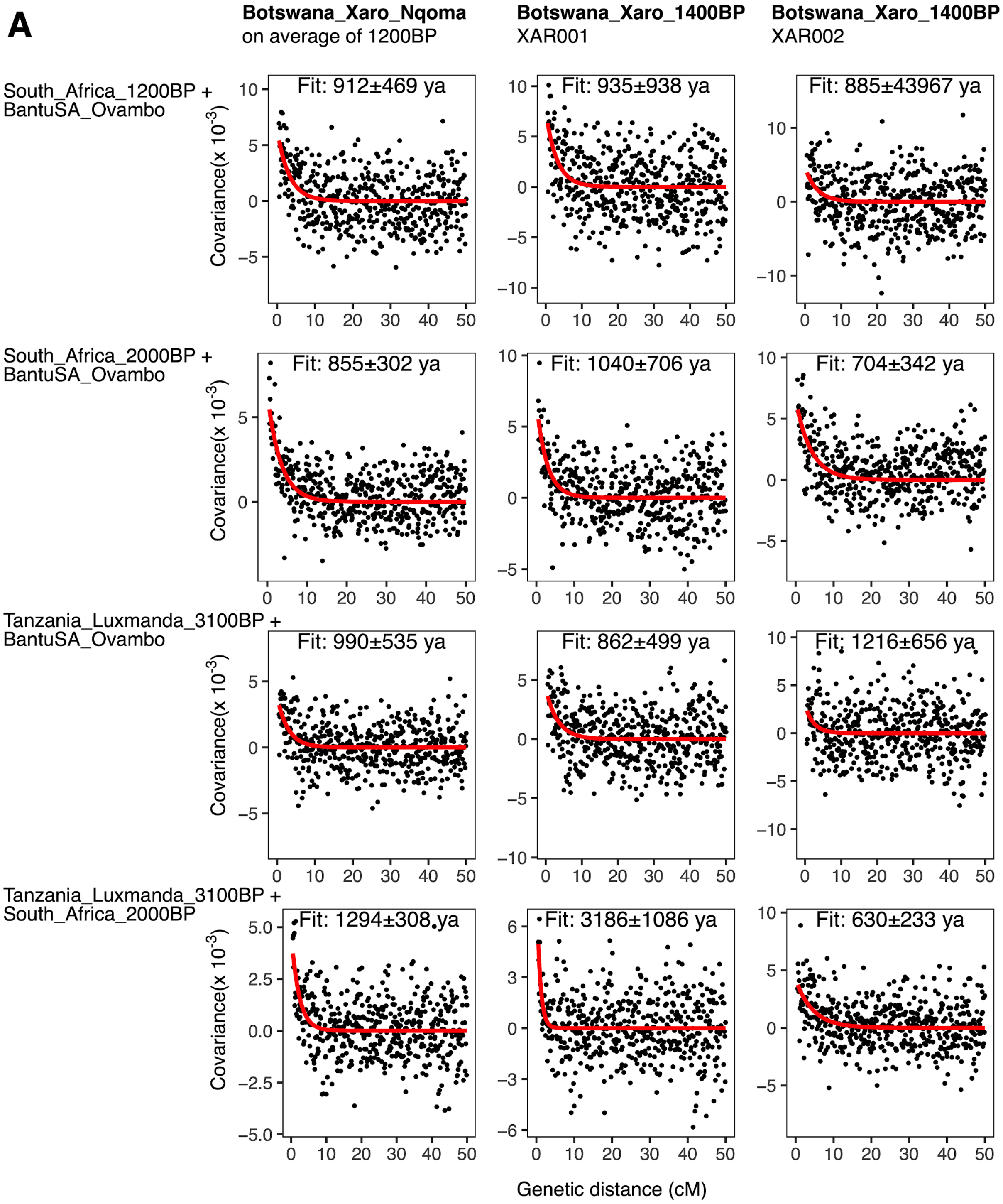
A
Botswana_Xaro_Nqoma
on average of 1200BP
Botswana_Xaro_1400BP
XAR001
Botswana_Xaro_1400BP
XAR002
South_Africa_1200BP +
BantuSA_Ovambo
South_Africa_2000BP +
BantuSA_Ovambo
Tanzania_Luxmanda_3100BP +
BantuSA_Ovambo
Tanzania_Luxmanda_3100BP +
South_Africa_2000BP
Fit: 912±469 ya
Fit: 935±938 ya
Fit: 885±43967 ya
Fit: 855±302 ya
Fit: 1040±706 ya
Fit: 704±342 ya
Fit: 990±535 ya
Fit: 862±499 ya
Fit: 1216±656 ya
Fit: 1294±308 ya
Fit: 3186±1086 ya
Fit: 630±233 ya
Covariance(x 10^-3)
Genetic distance (cM)

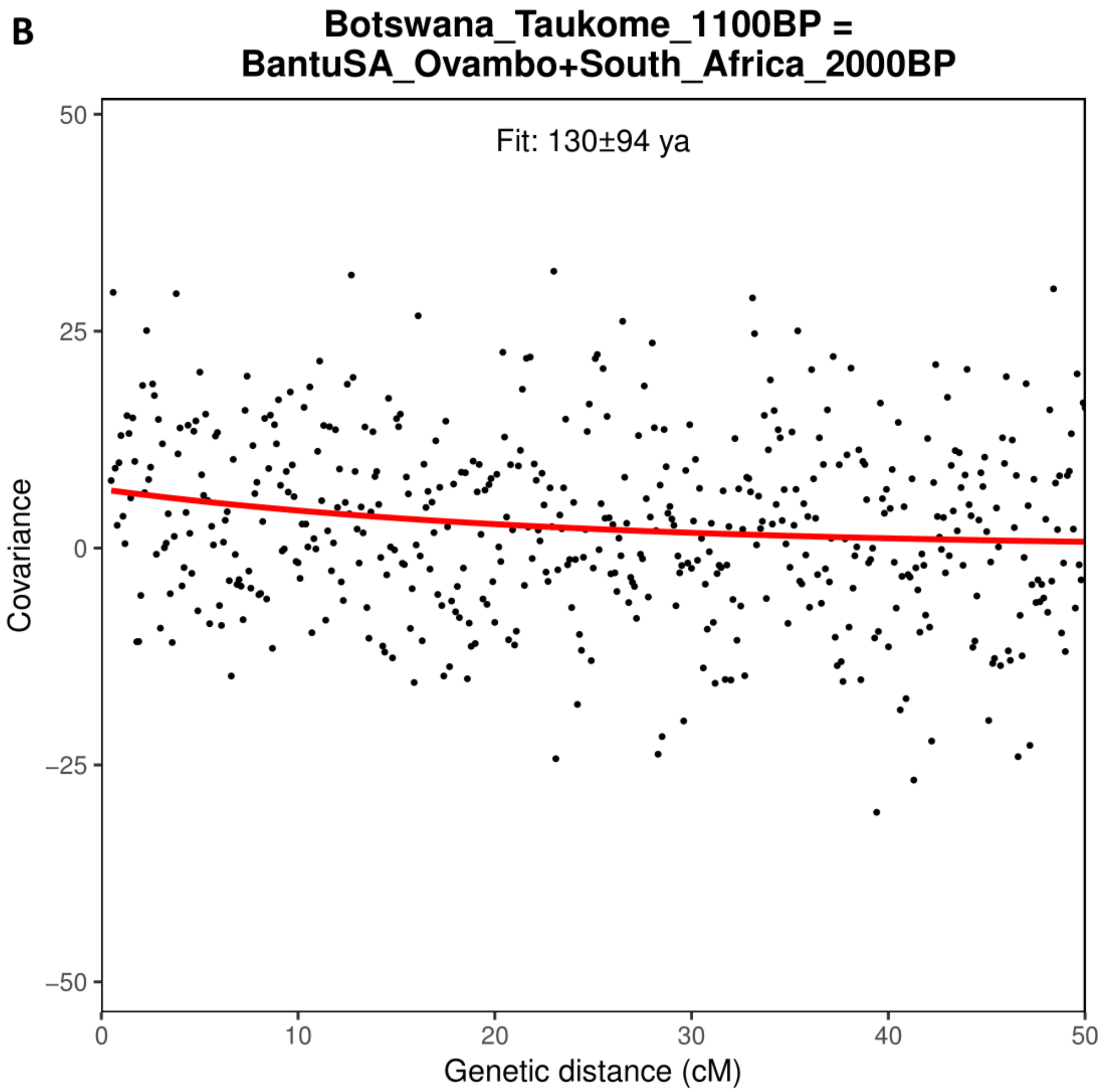

Figure S7. Dating the admixture between Bantu-related ancestry and southern African forager ancestry in ancient Botswana using DATES. In (A), for individuals from Xaro, Nqoma site, we dated four combinations of 2-way admixture from four sources: South_Africa_1200BP, South_Africa_2000BP, BantuSA_Ovambo and Tanzania_Luxmanda_3100BP, shown in different rows, in three ancient Botswana groups (one on grouped-level and the other two on individual-level) shown in different columns. The introduction of Bantu-related ancestry into southern African can be dated to on average of 2,000±400 years ago. The introduction of eastern African pastoralists can be dated into on average of 2500±300 years ago, slightly earlier than the time estimates of incoming Bantu-related ancestry in southern Africa. In (B), for individual Botswana_Taukome_1100BP from Taukome site, we dated the admixture between South_Africa_2000BP and BantuSA_Ovambo occured at 130±94 years ago before the death of this individual.

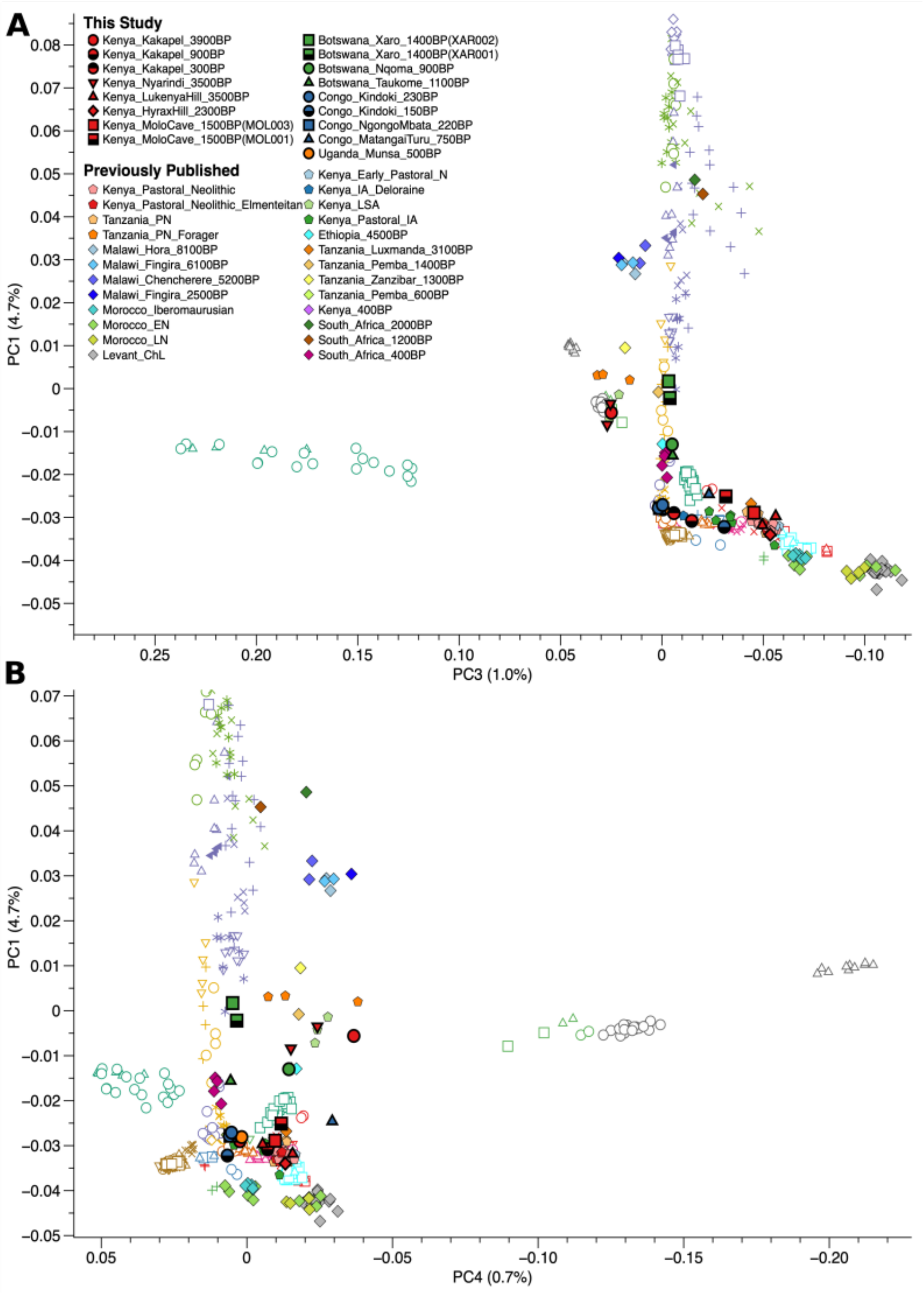

Figure S8. Illustrating genetic variations using more PCs, related to Figure 2 and Fig S2. (A) PC1 versus PC3. (B) PC1 versus PC4. Full legend of modern populations has been shown in Fig S1.

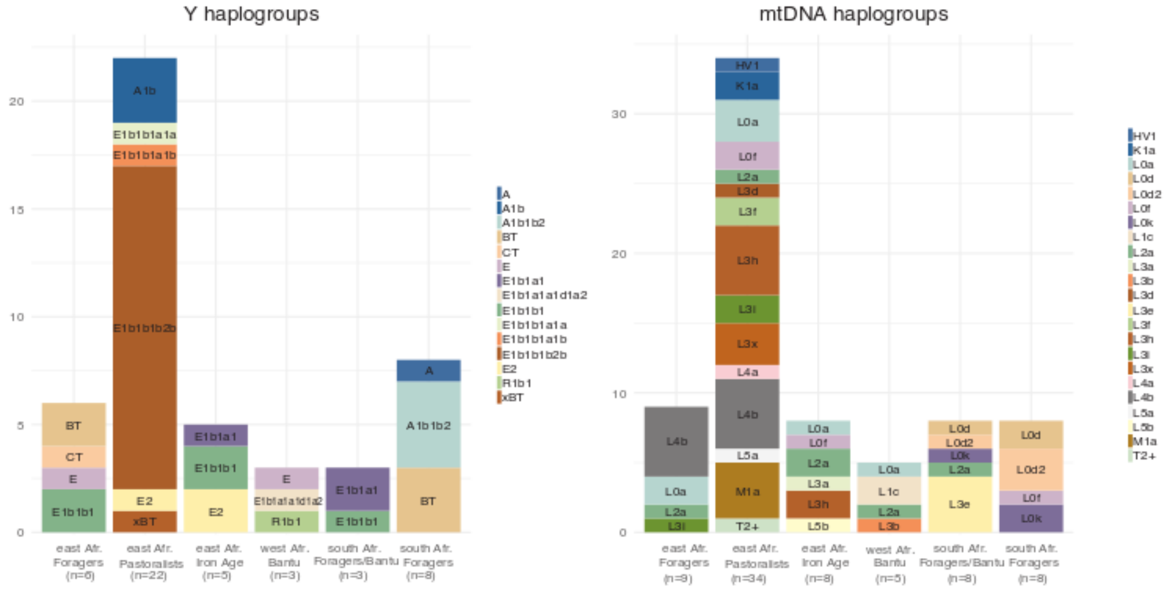

Figure S9. Distribution of mitochondrial and Y chromosome haplogroups in the ancient African genetic clusters. We show (A) the distribution of Y haplogroups in each genetic cluster, (B) distribution of mitochondrial haplogroups in each genetic cluster. The haplogroup information of every genetic cluster is detailed in Table S10.

REFERENCES AND NOTES

1. J. K. Pickrell, N. Patterson, C. Barbieri, F. Berthold, L. Gerlach, T. Güldemann, B. Kure, S. W. Mpoloka, H. Nakagawa, C. Naumann, M. Lipson, P.-R. Loh, J. Lachance, J. Mountain, C. D. Bustamante, B. Berger, S. A. Tishkoff, B. M. Henn, M. Stoneking, D. Reich, B. Pakendorf, The genetic prehistory of southern Africa. *Nat. Commun.* **3**, 1143 (2012).

2. C. M. Schlebusch, H. Malmström, T. Günther, P. Sjödin, A. Coutinho, H. Edlund, A. R. Munters, M. Vicente, M. Steyn, H. Soodyall, M. Lombard, M. Jakobsson, Southern African ancient genomes estimate modern human divergence to 350,000 to 260,000 years ago. *Science*. **358**, 652–655 (2017).

3. P. Skoglund, J. C. Thompson, M. E. Prendergast, A. Mittnik, K. Sirak, M. Hajdinjak, T. Salie, N. Rohland, S. Mallick, A. Peltzer, A. Heinze, I. Olalde, M. Ferry, E. Harney, M. Michel, K. Stewardson, J. I. Cerezo-Román, C. Chiumia, A. Crowther, E. Gomani-Chindebvu, A. O. Gidna, K. M. Grillo, I. T. Helenius, G. Hellenthal, R. Helm, M. Horton, S. López, A. Z. P. Mabulla, J. Parkington, C. Shipton, M. G. Thomas, R. Tibesasa, M. Welling, V. M. Hayes, D. J. Kennett, R. Ramesar, M. Meyer, S. Pääbo, N. Patterson, A. G. Morris, N. Boivin, R. Pinhasi, J. Krause, D. Reich, Reconstructing prehistoric African population structure. *Cell*. **171**, 59–71.e21 (2017).

4. M. E. Prendergast, M. Lipson, E. A. Sawchuk, I. Olalde, C. A. Ogola, N. Rohland, K. A. Sirak, N. Adamski, R. Bernardos, N. Broomandkhoshbacht, K. Callan, B. J. Culleton, L. Eccles, T. K. Harper, A. M. Lawson, M. Mah, J. Oppenheimer, K. Stewardson, F. Zalzala, S. H. Ambrose, G. Ayodo, H. L. Gates Jr, A. O. Gidna, M. Katongo, A. Kwekason, A. Z. P. Mabulla, G. S. Mudenda, E. K. Ndiema, C. Nelson, P. Robertshaw, D. J. Kennett, F. K. Manthi, D. Reich, Ancient DNA reveals a multistep spread of the first herders into sub-Saharan Africa. *Science* **365**, eaaw6275 (2019).

5. R. Pinhasi, D. Fernandes, K. Sirak, M. Novak, S. Connell, S. Alpaslan-Roodenberg, F. Gerritsen, V. Moiseyev, A. Gromov, P. Raczky, A. Anders, M. Pietrusewsky, G. Rollefson, M. Jovanovic, H. Trinhhoang, G. Bar-Oz, M. Oxenham, H. Matsumura, M. Hofreiter, Optimal ancient DNA yields from the inner ear part of the human petrous bone. *PLOS One*. **10**, e0129102 (2015).

6. M. G. Llorente, E. R. Jones, A. Eriksson, V. Siska, K. W. Arthur, J. W. Arthur, M. C. Curtis, J. T. Stock, M. Coltorti, P. Pieruccini, S. Stretton, F. Brock, T. Higham, Y. Park, M.

Hofreiter, D. G. Bradley, J. Bhak, R. Pinhasi, A. Manica, Ancient Ethiopian genome reveals extensive Eurasian admixture throughout the African continent. *Science*. **350**, 820–822 (2015).

7. T. Guldemann, A linguist's view: Khoe-Kwadi speakers as the earliest food-producers of southern Africa. *South. Afr. Humanit.* **20**, 93–132 (2008).
8. J. K. Pickrell, N. Patterson, P.-R. Loh, M. Lipson, B. Berger, M. Stoneking, B. Pakendorf, D. Reich, Ancient west Eurasian ancestry in southern and eastern Africa. *Proc. Natl. Acad. Sci. U.S.A.***111**, 2632–2637 (2014).
9. M. G. Llorente, E. R. Jones, A. Eriksson, V. Siska, K. W. Arthur, J. W. Arthur, M. C. Curtis, J. T. Stock, M. Coltorti, P. Pieruccini, S. Stretton, F. Brock, T. Higham, Y. Park, M. Hofreiter, D. G. Bradley, J. Bhak, R. Pinhasi, A. Manica, Ancient Ethiopian genome reveals extensive Eurasian admixture in Eastern Africa. *Science*. **350**, 820–822 (2015).
10. M. van de Loosdrecht, A. Bouzouggar, L. Humphrey, C. Posth, N. Barton, A. Aximu-Petri, B. Nickel, S. Nagel, E. H. Talbi, M. A. El Hajraoui, S. Amzazi, J.-J. Hublin, S. Pääbo, S. Schiffels, M. Meyer, W. Haak, C. Jeong, J. Krause, Pleistocene North African genomes link near Eastern and sub-Saharan African human populations. *Science* **360**, 548–552 (2018).
11. R. Fregel, F. L. Méndez, Y. Bokbot, D. Martín-Socas, M. D. Camalich-Massieu, J. Santana, J. Morales, M. C. Ávila-Arcos, P. A. Underhill, B. Shapiro, G. Wojcik, M. Rasmussen, A. E. R. Soares, J. Kapp, A. Sockell, F. J. Rodríguez-Santos, A. Mikdad, A. Trujillo-Mederos, C. D. Bustamante, Ancient genomes from north Africa evidence prehistoric migrations to the Maghreb from both the Levant and Europe. *Proc. Natl. Acad. Sci. U.S.A.***115**, 6774–6779 (2018).
12. I. Lazaridis, N. Patterson, A. Mittnik, G. Renaud, S. Mallick, K. Kirsanow, P. H. Sudmant, J. G. Schraiber, S. Castellano, M. Lipson, B. Berger, C. Economou, R. Bollongino, Q. Fu, K. I. Bos, S. Nordenfelt, H. Li, C. de Filippo, K. Prüfer, S. Sawyer, C. Posth, W. Haak, F. Hallgren, E. Fornander, N. Rohland, D. Delsate, M. Francken, J.-M. Guinet, J. Wahl, G. Ayodo, H. A. Babiker, G. Bailliet, E. Balanovska, O. Balanovsky, R. Barrantes, G. Bedoya, H. Ben-Ami, J. Bene, F. Berrada, C. M. Bravi, F. Brisighelli, G. B. J. Busby, F. Cali, M. Churnosov, D. E. C. Cole, D. Corach, L. Damba, G. van Driem, S. Dryomov, J.-M. Dugoujon, S. A. Fedorova, I. G. Romero, M. Gubina, M. Hammer, B. M. Henn, T. Hervig, U. Hodoglugil, A. R. Jha, S. Karachanak-Yankova, R. Khusainova, E. Khusnutdinova, R.

Kittles, T. Kivisild, W. Klitz, V. Kučinskas, A. Kushniarevich, L. Laredj, S. Litvinov, T. Loukidis, R. W. Mahley, B. Melegh, E. Metspalu, J. Molina, J. Mountain, K. Näkkäläjärvi, D. Nesheva, T. Nyambo, L. Osipova, J. Parik, F. Platonov, O. Posukh, V. Romano, F. Rothhammer, I. Rudan, R. Ruizbakiev, H. Sahakyan, A. Sajantila, A. Salas, E. B. Starikovskaya, A. Tarekegn, D. Toncheva, S. Turdikulova, I. Uktveryte, O. Utevska, R. Vasquez, M. Villena, M. Voevoda, C. A. Winkler, L. Yepiskoposyan, P. Zalloua, T. Zemunik, A. Cooper, C. Capelli, M. G. Thomas, A. Ruiz-Linares, S. A. Tishkoff, L. Singh, K. Thangaraj, R. Villems, D. Comas, R. Sukernik, M. Metspalu, M. Meyer, E. E. Eichler, J. Burger, M. Slatkin, S. Pääbo, J. Kelso, D. Reich, J. Krause, Ancient human genomes suggest three ancestral populations for present-day Europeans. *Nature*. **513**, 409–413 (2014).

13. S. Fan, D. E. Kelly, M. H. Beltrame, M. E. B. Hansen, S. Mallick, A. Ranciaro, J. Hirbo, S. Thompson, W. Beggs, T. Nyambo, S. A. Omar, D. W. Meskel, G. Belay, A. Froment, N. Patterson, D. Reich, S. A. Tishkoff, African evolutionary history inferred from whole genome sequence data of 44 indigenous African populations. *Genome Biol.***20**, 82 (2019).
14. S. Mallick, H. Li, M. Lipson, I. Mathieson, M. Gymrek, F. Racimo, M. Zhao, N. Chennagiri, S. Nordenfelt, A. Tandon, P. Skoglund, I. Lazaridis, S. Sankararaman, Q. Fu, N. Rohland, G. Renaud, Y. Erlich, T. Willems, C. Gallo, J. P. Spence, Y. S. Song, G. Poletti, F. Balloux, G. van Driem, P. de Knijff, I. G. Romero, A. R. Jha, D. M. Behar, C. M. Bravi, C. Capelli, T. Hervig, A. Moreno-Estrada, O. L. Posukh, E. Balanovska, O. Balanovsky, S. Karachanak-Yankova, H. Sahakyan, D. Toncheva, L. Yepiskoposyan, C. Tyler-Smith, Y. Xue, M. S. Abdullah, A. Ruiz-Linares, C. M. Beall, A. Di Rienzo, C. Jeong, E. B. Starikovskaya, E. Metspalu, J. Parik, R. Villems, B. M. Henn, U. Hodoglugil, R. Mahley, A. Sajantila, G. Stamatoyannopoulos, J. T. S. Wee, R. Khusainova, E. Khusnutdinova, S. Litvinov, G. Ayodo, D. Comas, M. F. Hammer, T. Kivisild, W. Klitz, C. A. Winkler, D. Labuda, M. Bamshad, L. B. Jorde, S. A. Tishkoff, W. S. Watkins, M. Metspalu, S. Dryomov, R. Sukernik, L. Singh, K. Thangaraj, S. Pääbo, J. Kelso, N. Patterson, D. Reich, The simons genome diversity project: 300 genomes from 142 diverse populations. *Nature*. **538**, 201–206 (2016).
15. G. Renaud, V. Slon, A. T. Duggan, J. Kelso, Schmutzi: Estimation of contamination and endogenous mitochondrial consensus calling for ancient DNA. *Genome Biol.***16**, 224 (2015).

16. Q. Fu, A. Mittnik, P. L. F. Johnson, K. Bos, M. Lari, R. Bollongino, C. Sun, L. Giemsch, R. Schmitz, J. Burger, A. M. Ronchitelli, F. Martini, R. G. Cremonesi, J. Svoboda, P. Bauer, D. Caramelli, S. Castellano, D. Reich, S. Pääbo, J. Krause, A revised timescale for human evolution based on ancient mitochondrial genomes. *Curr. Biol.* **23**, 553–559 (2013).
17. T. S. Korneliussen, A. Albrechtsen, R. Nielsen, ANGSD: Analysis of next generation sequencing data. *BMC Bioinformatics* **15**, 356 (2014).
18. D. H. Alexander, J. Novembre, K. Lange, Fast model-based estimation of ancestry in unrelated individuals. *Genome Res.* **19**, 1655–1664 (2009).
19. W. Haak, I. Lazaridis, N. Patterson, N. Rohland, S. Mallick, B. Llamas, G. Brandt, S. Nordenfelt, E. Harney, K. Stewardson, Q. Fu, A. Mittnik, E. Bánffy, C. Economou, M. Francken, S. Friederich, R. G. Pena, F. Hallgren, V. Khartanovich, A. Khokhlov, M. Kunst, P. Kuznetsov, H. Meller, O. Mochalov, V. Moiseyev, N. Nicklisch, S. L. Pichler, R. Risch, M. A. Rojo Guerra, C. Roth, A. Szécsényi-Nagy, J. Wahl, M. Meyer, J. Krause, D. Brown, D. Anthony, A. Cooper, K. W. Alt, D. Reich, Massive migration from the steppe was a source for Indo-European languages in Europe. *Nature*. **522**, 207–211 (2015).
20. B. M. Henn, L. R. Botigué, S. Gravel, W. Wang, A. Brisbin, J. K. Byrnes, K. Fadhlaoui-Zid, P. A. Zalloua, A. Moreno-Estrada, J. Bertranpetit, C. D. Bustamante, D. Comas, Genomic ancestry of North Africans supports back-to-Africa migrations. *PLOS Genet.* **8**, e1002397 (2012).
21. R. Kuper, S. Kröpelin, Climate-controlled holocene occupation in the sahara: Motor of Africa's evolution. *Science* **313**, 803–807 (2006).
22. A. G. Morris, The myth of the east african `Bushmen'. *South Afr. Archaeol. Bull.*, **58**, 85–90 (2003).
23. T. Güldemann, Greenberg's" case" for Khoisan: The morphological evidence, in *Problems of Linguistic-Historical Reconstruction in Africa*, D. Ibriszimov, Ed. (Köln: Rüdiger Köppe, 2008), pp. 123–153.
24. É. Harney, H. May, D. Shalem, N. Rohland, S. Mallick, I. Lazaridis, R. Sarig, K. Stewardson, S. Nordenfelt, N. Patterson, I. Hershkovitz, D. Reich, Ancient DNA from chalcolithic israel reveals the role of population mixture in cultural transformation. *Nat. Commun.* **9**, 3336 (2018).

25. S. H. Ambrose, Chronology of the later stone age and food production in east africa. *J. Archaeol. Sci.***25**, 377–392 (1998).
26. P. J. Lane, The "Moving Frontier" and the transition to food production in Kenya. *Azania* **39**, 243–264 (2004).
27. D. Gifford-Gonzalez, Early pastoralists in east africa: Ecological and social dimensions. *J. Anthropol. Archaeol.* **17**, 166–200 (1998).
28. M. E. Prendergast, K. K. Mutundu, Late holocene archaeological faunas in east Africa: Ethnographic analogues and interpretive challenges. *Documenta Archaeobiologiae*. **7**, 203–232 (2009).
29. J. C. Onyango-Abuje, Crescent island: A preliminary report on excavations at an east african neolithic site. *Azania Archaeol. Res. Africa* **12**, 147–159 (1977).
30. D. P. Gifford, G. L. Isaac, C. M. Nelson, Evidence for predation and pastoralism at prolonged drift: A pastoral neolithic site in kenya. *Azania* **15**, 57–108 (1980).
31. A. B. Smith, Keeping people on the periphery: The ideology of social hierarchies between hunters and herders. *J. Anthropol. Archaeol.* **17**, 201–215 (1998).
32. V. Bajić, C. Barbieri, A. Hübner, T. Güldemann, C. Naumann, L. Gerlach, F. Berthold, H. Nakagawa, S. W. Mpoloka, L. Roewer, J. Purps, M. Stoneking, B. Pakendorf, Genetic structure and sex-biased gene flow in the history of southern african populations. *Am. J. Phys. Anthropol.***167**, 656–671 (2018).
33. B. A. Ogot, *History of the Southern Luo. Volume 1. Migration and Settlement, 1500-1900* (East African Publishing House, 1967).
34. P. Robertshaw, Munsa earthworks: A preliminary report on recent excavations. *Azani Arch. Res. Africa* **32**, 1–20 (1997).
35. S. A. Tishkoff, M. K. Gonder, B. M. Henn, H. Mortensen, A. Knight, C. Gignoux, N. Fernandopulle, G. Lema, T. B. Nyambo, U. Ramakrishnan, F. A. Reed, J. L. Mountain, History of click-speaking populations of africa inferred from mtDNA and Y chromosome genetic variation. *Mol. Biol. Evol.* **24**, 2180–2195 (2007).
36. C. M. Schlebusch, T. Naidoo, H. Soodyall, SNaPshot minisequencing to resolve mitochondrial macro-haplogroups found in Africa. *Electrophoresis* **30**, 3657–3664 (2009).
37. B. M. Henn, C. Gignoux, A. A. Lin, P. J. Oefner, P. Shen, R. Scozzari, F. Cruciani, S. A. Tishkoff, J. L. Mountain, P. A. Underhill, Y-chromosomal evidence of a pastoralist

migration through tanzania to southern Africa. *Proc. Natl. Acad. Sci. U.S.A.***105**, 10693–10698 (2008).

38. N. Isern, J. Fort, Assessing the importance of cultural diffusion in the Bantu spread into southeastern Africa. *PLOS One* **14**, e0215573 (2019).
39. G. Breton, C. M. Schlebusch, M. Lombard, P. Sjödin, H. Soodyall, M. Jakobsson, Lactase persistence alleles reveal partial east african ancestry of southern african Khoe pastoralists. *Curr. Biol.* **24**, 852–858 (2014).
40. E. Macholdt, V. Lede, C. Barbieri, S. W. Mpoloka, H. Chen, M. Slatkin, B. Pakendorf, M. Stoneking, Tracing pastoralist migrations to southern Africa with lactase persistence alleles. *Curr. Biol.* **24**, 875–879 (2014).
41. N. Sepúlveda, A. Manjurano, S. G. Campino, M. Lemnge, J. Lusingu, R. Olomi, K. A. Rockett, C. Hubbart, A. Jeffreys, K. Rowlands, T. G. Clark, E. M. Riley, C. J. Drakeley; MalariaGEN Consortium, Malaria host candidate genes validated by association with current, recent, and historical measures of transmission intensity. *J Infect Dis.* **216**, 45–54 (2017).
42. K. A. Murphy, A meal on the hoof or wealth in the kraal? Stable isotopes at Kgaswe and Taukome in eastern Botswana. *Int. J. Osteoarchaeol.* **21**, 591–601 (2011).
43. G. Turner, Early iron age herders in northwestern Botswana: The faunal evidence. *Botsw. Notes Rec.* **19**, 7–23 (1987).
44. J. K. Thornton, L. Heywood, Afro-Latino Voices, *Narratives from the Early Modern Ibero-Atlantic World, 1550-1812*, K. J. McKnight, L. J. Garofalo, Eds. (Hackett Publishing, 2009).
45. J. E. Yellen, Barbed bone points: Tradition and continuity in Saharan and sub-Saharan Africa. *African Arch. Rev.* **15**, 173–198 (1998).
46. B. Keding, Middle holocene fisher-hunter-gatherers of lake turkana in Kenya and their cultural connections with the north: The pottery. *J. African Arch.* **15**, 42–76 (2017).
47. A. Crowther, M. E. Prendergast, D. Q. Fuller, N. Boivin, Subsistence mosaics, forager-farmer interactions, and the transition to food production in eastern Africa. *Quat. Int.* **489**, 101–120 (2018).
48. P. Mitchell, Early farming communities of southern and south-central Africa, in *The Oxford Handbook of African Archaeology*, P. Mitchell, P. Lane, Eds. (Oxford Univ. Press, 2013), pp. 657– 670.

49. S. A. Tishkoff, F. A. Reed, F. R. Friedlaender, C. Ehret, A. Ranciaro, A. Froment, J. B. Hirbo, A. A. Awomoyi, J.-M. Bodo, O. Doumbo, M. Ibrahim, A. T. Juma, M. J. Kotze, G. Lema, J. H. Moore, H. Mortensen, T. B. Nyambo, S. A. Omar, K. Powell, G. S. Pretorius, M. W. Smith, M. A. Thera, C. Wambebe, J. L. Weber, S. M. Williams, The genetic structure and history of Africans and African Americans. *Science* **324**, 1035–1044 (2009).
50. P. J. Reimer, E. Bard, A. Bayliss, J. Warren Beck, P. G. Blackwell, C. B. Ramsey, C. E. Buck, H. Cheng, R. Lawrence Edwards, M. Friedrich, P. M. Grootes, T. P. Guilderson, H. Haflidason, I. Hajdas, C. Hatté, T. J. Heaton, D. L. Hoffmann, A. G. Hogg, K. A. Hughen, K. Felix Kaiser, B. Kromer, S. W. Manning, M. Niu, R. W. Reimer, D. A. Richards, E. Marian Scott, J. R. Southon, R. A Staff, C. S. M. Turney, J. van der Plicht, IntCal13 and Marine13 radiocarbon age calibration curves 0–50,000 years cal BP. *Radiocarbon*. **55**, 1869–1887 (2013).
51. C. Bronk Ramsey, T. F. G. Higham, F. Brock, D. Baker, P. Ditchfield, Radiocarbon dates from the Oxford AMS system: *Archaeometry* Datelist 33. *Archaeometry* **51**, 323–349 (2009).
52. J. Dabney, M. Knapp, I. Glocke, M.-T. Gansauge, A. Weihmann, B. Nickel, C. Valdiosera, N. García, S. Pääbo, J.-L. Arsuaga, M. Meyer, Complete mitochondrial genome sequence of a middle pleistocene cave bear reconstructed from ultrashort DNA fragments. *Proc. Natl. Acad. Sci. U.S.A.* **110**, 15758–15763 (2013).
53. N. Rohland, E. Harney, S. Mallick, S. Nordenfelt, D. Reich, Partial uracil–DNA–glycosylase treatment for screening of ancient DNA. *Philos. Trans. R. Soc. Lond. B Biol. Sci.* **370**, 20130624 (2015).
54. Q. Fu, M. Hajdinjak, O. T. Moldovan, S. Constantin, S. Mallick, P. Skoglund, N. Patterson, N. Rohland, I. Lazaridis, B. Nickel, B. Viola, K. Prüfer, M. Meyer, J. Kelso, D. Reich, S. Pääbo, An early modern human from Romania with a recent neanderthal ancestor. *Nature* **524**, 216–219 (2015).
55. A. Peltzer, G. Jäger, A. Herbig, A. Seitz, C. Kniep, J. Krause, K. Nieselt, EAGER: Efficient ancient genome reconstruction. *Genome Biol.* **17**, 60 (2016).
56. M. Schubert, S. Lindgreen, L. Orlando, AdapterRemoval v2: Rapid adapter trimming, identification, and read merging. *BMC. Res. Notes* **9**, 88 (2016).
57. H. Li, R. Durbin, Fast and accurate short read alignment with Burrows–Wheeler transform. *Bioinformatics* **25**, 1754–1760 (2009).

58. G. Jun, M. K. Wing, G. R. Abecasis, H. M. Kang, An efficient and scalable analysis framework for variant extraction and refinement from population-scale DNA sequence data. *Genome Res.* **25**, 918–925 (2015).
59. H. Weissensteiner, D. Pacher, A. Kloss-Brandstätter, L. Forer, G. Specht, H.-J. Bandelt, F. Kronenberg, A. Salas, S. Schönherr, HaploGrep 2: Mitochondrial haplogroup classification in the era of high-throughput sequencing. *Nucleic Acids Res.* **44**, W58–W63 (2016).
60. D. Vianello, F. Sevini, G. Castellani, L. Lomartire, M. Capri, C. Franceschi, HAPLOFIND: A new method for high-throughput mtDNA haplogroup assignment. *Hum. Mutat.* **34**, 1189–1194 (2013)
61. M. Kearse, R. Moir, A. Wilson, S. Stones-Havas, M. Cheung, S. Sturrock, S. Buxton, A. Cooper, S. Markowitz, C. Duran, T. Thierer, B. Ashton, P. Meintjes, A. Drummond, Geneious basic: An integrated and extendable desktop software platform for the organization and analysis of sequence data. *Bioinformatics* **28**, 1647–1649 (2012).
62. G. David Poznik, Identifying Y-chromosome haplogroups in arbitrarily large samples of sequenced or genotyped men. *bioRxiv,* 088716 (2016).
63. J. M. Monroy Kuhn, M. Jakobsson, T. Günther, Estimating genetic kin relationships in prehistoric populations. *PLOS One* **13**, e0195491 (2018).
64. N. Patterson, P. Moorjani, Y. Luo, S. Mallick, N. Rohland, Y. Zhan, T. Genschoreck, T. Webster, D. Reich, Ancient admixture in human history. *Genetics* **192**, 1065–1093 (2012).
65. I. Lazaridis, D. Nadel, G. Rollefson, D. C. Merrett, N. Rohland, S. Mallick, D. Fernandes, M. Novak, B. Gamarra, K. Sirak, S. Connell, K. Stewardson, E. Harney, Q. Fu, G. Gonzalez-Fortes, E. R. Jones, S. A. Roodenberg, G. Lengyel, F. Bocquentin, B. Gasparian, J. M. Monge, M. Gregg, V. Eshed, A.-S. Mizrahi, C. Meiklejohn, F. Gerritsen, L. Bejenaru, M. Blüher, A. Campbell, G. Cavalleri, D. Comas, P. Froguel, E. Gilbert, S. M. Kerr, P. Kovacs, J. Krause, D. McGettigan, M. Merrigan, D. A. Merriwether, S. O'Reilly, M. B. Richards, O. Semino, M. Shamoon-Pour, G. Stefanescu, M. Stumvoll, A. Tönjes, A. Torroni, J. F. Wilson, L. Yengo, N. A. Hovhannisyan, N. Patterson, R. Pinhasi, D. Reich, Genomic insights into the origin of farming in the ancient near east. *Nature*. **536**, 419–424 (2016).
66. N. Patterson, A. L. Price, D. Reich, Population structure and eigenanalysis. *PLOS Genet.* **2**, e190 (2006).

67. V. M. Narasimhan, N. Patterson, P. Moorjani, I. Lazaridis, M. Lipson, S. Mallick, N. Rohland, R. Bernardos, A. M. Kim, N. Nakatsuka, I. Olalde, A. Coppa, J. Mallory, V. Moiseyev, J. Monge, L. M. Olivieri, N. Adamski, N. Broomandkhoshbacht, F. Candilio, O. Cheronet, B. J. Culleton, M. Ferry, D. Fernandes, B. Gamarra, D. Gaudio, M. Hajdinjak, É. Harney, T. K. Harper, D. Keating, A. M. Lawson, M. Michel, M. Novak, J. Oppenheimer, N. Rai, K. Sirak, V. Slon, K. Stewardson, Z. Zhang, G. Akhatov, A. N. Bagashev, B. Baitanayev, G. L. Bonora, T. Chikisheva, A. Derevianko, E. Dmitry, K. Douka, N. Dubova, A. Epimakhov, S. Freilich, D. Fuller, A. Goryachev, A. Gromov, B. Hanks, M. Judd, E. Kazizov, A. Khokhlov, E. Kitov, E. Kupriyanova, P. Kuznetsov, D. Luiselli, F. Maksudov, C. Meiklejohn, D. Merrett, R. Micheli, O. Mochalov, Z. Muhammed, S. Mustafokulov, A. Nayak, R. M. Petrovna, D. Pettener, R. Potts, D. Razhev, S. Sarno, K. Sikhymbaeva, S. M. Slepchenko, N. Stepanova, S. Svyatko, S. Vasilyev, M. Vidale, D. Voyakin, A. Yermolayeva, A. Zubova, V. S. Shinde, C. Lalueza-Fox, M. Meyer, D. Anthony, N. Boivin, K. Thangaraj, D. J. Kennett, M. Frachetti, R. Pinhasi, D. Reich, The genomic formation of South and Central Asia. bioRxiv 292581 [**Preprint**] (31 March 2018).
68. M. Feldman, D. M. Master, R. A. Bianco, M. Burri, P. W. Stockhammer, A. Mittnik, A. J. Aja, C. Jeong, J. Krause, Ancient DNA sheds light on the genetic origins of early iron age philistines. *Sci. Adv.*. **5**, eaax0061 (2019).
69. C. A. Tryon, I. Crevecoeur, J. T. Faith, R. Ekshtain, J. Nivens, D. Patterson, E. N. Mbua, F. Spoor, Late pleistocene age and archaeological context for the hominin calvaria from GvJm-22 (Lukenya Hill, Kenya). *Proc. Natl. Acad. Sci. U.S.A.* **112**, 2682–2687 (2015).
70. F. Marshall, R. E. B. Reid, S. Goldstein, M. Storozum, A. Wreschnig, L. Hu, P. Kiura, R. Shahack-Gross, S. H. Ambrose, Ancient herders enriched and restructured African grasslands. *Nature* **561**, 387–390 (2018).
71. C. M. Nelson, J. Kimegich, in *Origin and Early Development of Food – Producing Cultures in North-Eastern Africa* (Poznan Archaeological Museum, 1984) pp. 481–487.
72. S. H. Ambrose, M. J. DeNiro, Reconstruction of African human diet using bone collagen carbon and nitrogen isotope ratios. *Nature* **319**, 321–324 (1986).
73. E. A. Sawchuk, thesis, University of Toronto (2017).
74. L. A. Schepartz, thesis, University of Michigan (1987).

75. M. D. Leakey, L. S. B. Leakey, P. M. Game, A. J. H. Goodwin, Report on the excavations at Hyrax Hill, Nakuru, Kenya Colony, 1937–1938. *Trans. R. Soc. S. Afr.* **30**, 271–409 (1943).
76. E. A. Hildebrand, K. M. Grillo, E. A. Sawchuk, S. K. Pfeiffer, L. B. Conyers, S. T. Goldstein, A. C. Hill, A. Janzen, C. E. Klehm, M. Helper, P. Kiura, E. Ndiema, C. Ngugi, J. J. Shea, H. Wang, A monumental cemetery built by eastern Africa's first herders near Lake Turkana, Kenya. *Proc. Natl. Acad. Sci. U.S.A.* **115**, 8942–8947 (2018).
77. H. Field, The University of California African expedition: II, Sudan and Kenya. *Am. Anthropol.* **51**, 72–84 (1949).
78. W. E. Owen, 76. The Early Smithfield culture of Kavirondo (Kenya) and South Africa. *Man.* **41**, 115 (1941).
79. J. L. Buckberry, A. T. Chamberlain, Age estimation from the auricular surface of the ilium: A revised method. *Am. J. Phys. Anthropol.* **119**, 231–239 (2002).
80. E. A. DiGangi, J. D. Bethard, E. H. Kimmerle, L. W. Konigsberg, A new method for estimating age-at-death from the first rib. *Am. J. Phys. Anthropol.* **138**, 164–176 (2009).
81. M. Trotter, R. R. Peterson, Weight of the skeleton during postnatal development. *Am. J. Phys. Anthropol.* **33**, 313–323 (1970).
82. E. C. Lanning, Ancient earthworks in western Uganda. *Uganda J.* **17**, 51–62 (1953).
83. P. Robertshaw, The age and function of ancient earthworks of western Uganda. *Uganda J.* **47**, 20–33 (2001).
84. E. C. Lanning, The munsa earthworks. *Uganda J.* **19**, 177–182 (1955).
85. L. Iles, P. Robertshaw, R. Young, A furnace and associated ironworking remains at Munsa, Uganda. *Azania Arch. Res. Africa* **49**, 45–63 (2014).
86. R. L. Tantala, thesis, University of Wisconsin, Madison (1989).
87. P. Robertshaw, The ancient earthworks of western Uganda: Capital sites of a Cwezi empire? *Uganda J.* **48**, 17–32 (2002).
88. J. Mercader, M. D. Garralda, O. M. Pearson, R. C. Bailey, Eight hundred-year-old human remains from the Ituri tropical forest, democratic republic of congo: The rock shelter site of Matangai Turu northwest. *Am. J. Phys. Anthropol.* **115**, 24–37 (2001).
89. J. Mercader, F. Runge, L. Vrydaghs, H. Doutrelepont, C. E. N. Ewango, J. Juan-Tresseras, Phytoliths from archaeological sites in the tropical forest of Ituri, democratic republic of congo. *Quatern. Res.* **54**, 102–112 (2000).

90. J. Mercader, S. Rovira, P. Gómez-Ramos, Forager-farmer interaction and ancient iron metallurgy in the Ituri rainforest, democratic republic of congo. *Azania Arch. Res. Africa*. **35**, 107–122 (2000).
91. B. Clist, E. Cranshof, G.-M. de Schryver, D. Herremans, K. Karklins, I. Matonda, C. Polet, A. Sengeløv, F. Steyaert, C. Verhaeghe, K. Bostoen, The elusive archaeology of kongo urbanism: The case of kindoki, Mbanza Nsundi (Lower Congo, DRC). *African Arch. Rev.* **32**, 369–412 (2015).
92. B. Clist, E. Cranshof, P. de Maret, M. Kaumba Mazanga, R. Kidebua, I. Matonda, A. Nkanza Lutayi, J. Yogolelo, in *Une Archéologie des Provinces Septentrionales du Royaume Kongo* (Archaeopress, 2018), pp. 135–164.
93. B. Clist, N. Nikis, P. de Maret, in *Une Archéologie des Provinces Septentrionales du Royaume Kongo*, (Archaeopress, 2018), pp. 243–295.
94. J. K. Thornton, in *The Kongo Kingdom: The Origins, Dynamics and Cosmopolitan Culture of an African Polity* (Cambridge Univ. Press, 2018), pp. 17–41.
95. C. Polet, in *Une archéologie des Provinces Septentrionales du Royaume Kongo,* (Archeopress, 2018), pp. 401–438.
96. C. Polet, B.-O. Clist, K. Bostoen, Étude des restes humains de Kindoki (République démocratique du Congo, fin XVIIe–Début XIXe siècle). *Bull. Mém. Soc. Anthropol. Paris* **30**, 70–89 (2018).
97. C. Verhaeghe, B.-O. Clist, C. Fontaine, K. Karklins, K. Bostoen, W. De Clercq, Shell and glass beads from the tombs of Kindoki, Mbanza Nsundi, lower congo. *Beads J. Soc. Bead Res.*. **26**, 23–34 (2014).
98. P. Dubrunfaut, B. Clist, in *Une Archéologie des Provinces Septentrionales du Royaume Kongo,* (Archaeopress, 2018), pp. 359–368.
99. K. Karklins, B. Clist, in *Une Archéologie des Provinces Septentrionales du Royaume Kongo,* (Archaeopress, 2018), pp. 337–348.
100. B. Clist, E. Cranshof, G.-M. de Schryver, D. Herremans, K. Karklins, I. Matonda, F. Steyaert, K. Bostoen, African-European contacts in the Kongo Kingdom (Sixteenth-eighteenth centuries): New archaeological insights from Ngongo Mbata (Lower Congo, DRC). *Int. J. Hist. Archaeol.* **19**, 464–501 (2015).

101. B. Clist, E. Cranshof, M. Kaumba Mazanga, I. Matonda Sakala, A. Nkanza Lutayi, J. Yogolelo, in *Une Archéologie des Provinces Septentrionales du Royaume Kongo* (Archaeopress, 2018), pp. 71–132.
102. M. Bequaert, Fouille d'un cimetière du XVIIe siècle au Congo Belge. *L'Antiquité Classique* **9**, 127–128 (1940).
103. E. Kose, New light on ironworking groups along the middle Kavango in northern Namibia. *South African Arch. Bull.* **64**, 130–147 (2009).
104. M. N. Mosothwane, Dietary stable carbon isotope signatures of the early iron age inhabitants of Ngamiland. *Botsw. Notes Rec.* **43**, 115–129 (2011).
105. E. N. Wilmsen, A. C. Campbell, G. A. Brook, L. H. Robbins, M. Murphy, Mining and moving specular haematite in Botswana, ca. 200–1300 AD, in *The World of Iron* (Archetype, 2013), pp. 33–45.
106. E. N. Wilmsen, Nqoma: An abridged review. *Botsw. Notes Rec.* **43**, 95–114 (2011).
107. J. R. Denbow, E. N. Wilmsen, Iron age pastoralist settlements in Botswana. *S. Afr. J. Sci.* **79**, 405–407 (1983).
108. J. Denbow, thesis, Indiana University (1983).
109. T. N. Huffman, *Handbook to the Iron Age* (Univ. of KwaZulu-Natal Press, 2007).
